# Computational Insights into the Activation Mechanism of CXCR4: Implications for the Design of Small Molecule Agonists

**DOI:** 10.1021/jacs.6c01087

**Published:** 2026-05-13

**Authors:** Jiao Zhou, Xiang Liu, Yan Xu, Alejandro Cruz, Jing An, Arieh Warshel, Ziwei Huang

**Affiliations:** † School of Chemistry and Chemical Engineering, 71237 Guangdong Pharmaceutical University, Zhongshan 528458, China; ‡ Ciechanover Institute of Precision and Regenerative Medicine, School of Life and Health Sciences, Chinese University of Hong Kong, Shenzhen 518172, China; § Department of Medicine, Division of Infectious Diseases and Global Public Health, School of Medicine, 407605 University of California at San Diego, La Jolla, San Diego, California 92037, United States; ∥ Departament d’Enginyeria Química (EQ), ETSEIB, 16767 Universitat Politècnica de Catalunya - BarcelonaTech (UPC), Campus Sud, Edif. PG, Av. Diagonal, 647, Barcelona 08028, Spain; ⊥ Department of Chemistry, 5116 University of Southern California, Los Angeles, California 90089, United States

## Abstract

G protein-coupled receptors (GPCRs) are transmembrane proteins that mediate diverse signaling functions, making them important therapeutic targets. The chemokine receptor CXCR4, a GPCR, plays multifaceted roles in both normal physiological and pathological processes. Here, we constructed conformational free energy profiles of CXCR4 activation using Targeted Molecular Dynamics (TMD) and Molecular Dynamics (MD) simulations combined with our refined Coarse-Grained (CG) model for membrane proteins. The simulations revealed that CXCR4 activation involves three distinct transition states. TS1, which exhibits the highest activation energy barrier, primarily involves conformational changes in intracellular loop 3 (ICL3) and the prerearrangement of transmembrane helices TM5 and TM6. Additionally, the stabilization of the specific active conformations of W94 and E288 within the CXCR4 active site was found to reduce the activation energy barriers of TS2 and TS3, respectively. Alanine scanning further revealed the dynamic roles of other residues whose putative crucial role in CXCR4 activation had previously been postulated, transitioning from stabilizing the inactive state to facilitating activation in later stages. Guided by these computational insights, we designed a small-molecule compound, HL82624, using a dual-moiety strategy combining the first two amino acids of SDF-1α (Lys and Pro) with the CXCR4 antagonist HF51116. Competitive binding and cell migration assays showed that HL82624 binds to CXCR4 and effectively triggers cell migration, confirming its activity as a CXCR4 agonist. Taken together, this study provides mechanistic insight into CXCR4 activation and a computational strategy for the rational design of small-molecule CXCR4 agonists.

## Graphical abstract

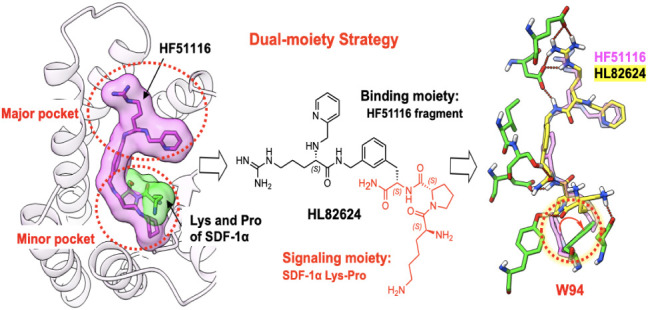

## Introduction

The chemokine receptor CXCR4, a member of the G-protein-coupled receptor (GPCR) superfamily, plays a pivotal role in numerous physiological and pathological processes, including HIV entry,^1,2^ cancer metastasis,^3,4^ inflammation,^5–7^ and stem cell trafficking.^8^ These multifaceted roles are mediated through the SDF-1α/CXCR4 signaling axis. Our laboratory has studied synthetic CXCR4-targeted ligands for almost three decades and developed many peptide and small-molecule CXCR4 antagonists with potent activity.^9–16^ While most drug discovery efforts to date have centered on CXCR4 antagonists,^17,18^ leading to the development of clinically approved small-molecule drugs such as AMD3100 ^19^ and AMD070,^20^ the development of CXCR4 agonists is relatively less explored.^21–23^ In recent years we have reported the promising therapeutic potential of CXCR4 agonists, particularly for neurodegenerative diseases.^23,24^ A synthetic peptide CXCR4 agonist, SDV1a, designed with a dual-moiety strategy by us, effectively guides neural stem cells (NSCs) to pathological sites of the central nervous system (CNS), activating repair pathways and promoting tissue regeneration in mouse models of neurodegenerative diseases. Unlike native SDF-1α, which can trigger undesirable inflammatory responses, SDV1a was engineered to contain a strong pure binding motif (from the first 21 N-terminal residues in D-chirality of vMIP-II,^25^ a CXCR4 antagonist) linked to a signaling motif (from the first 8 N-terminal residues of SDF-1α) devoid of inflammatory sequences. This design not only elicited more extensive and sustained NSC migration and distribution but also suppressed host inflammation, thereby inducing predominantly reparative gene expression. More recently, we showed that SDV1a could enhance widespread stem cell neuroprotection in a mouse model of amyotrophic lateral sclerosis (ALS).^24^ These findings demonstrate the potential value of SDV1a as a CXCR4 agonist for developing new therapeutics for neurodegeneration.

Understanding the structural mechanisms underlying CXCR4 activation is critical for the rational design and optimization of agonists. CXCR4 activation is a highly complex, multistep process involving ligand binding, receptor conformational changes, and coupling with G proteins.^26–28^ Recent studies have identified the rearrangement of the Proline-Isoleucine-Phenylalanine (PIF) motif as a crucial step in CXCR4 activation, and the important role of G protein precoupling in facilitating this process.^29^ Despite these advances, the molecular details of SDF-1α-mediated activation within the receptor’s active site remain poorly understood, presenting a significant barrier to the development of new agonists. A deeper examination of the structural transitions and activation pathways of CXCR4, particularly the events occurring within the active site, could enable the design of small-molecule agonists and expand the therapeutic possibilities for CXCR4-targeted treatments in regenerative medicine and beyond.

In our recent work, we leveraged cryoelectron microscopy (cryo-EM) to resolve the antagonist-bound structures of CXCR4 in complex with AMD3100, AMD070, and HF51116,^30^ as well as the active-state structures of CXCR4 bound to the synthetic agonists SDV1a and SDVX1 and coupled with G proteins.^24^ By comparing the inactive and active states of CXCR4, we identified significant conformational changes in residues W94 and E288 within the ligand-binding pocket (Figure 1), which might function as key molecular switches initiating activation signals. In addition to these local changes, we observed a recurring structural feature in active-state CXCR4 structures: a cholesterol molecule bound on the outer sides of TM2 and TM4 within the transmembrane region, whereas such cholesterol is absent in antagonist-bound structures (Figure S1). This consistent structural observation further motivated us to investigate its potential for helping modulate CXCR4 activation.

**Figure 1:**
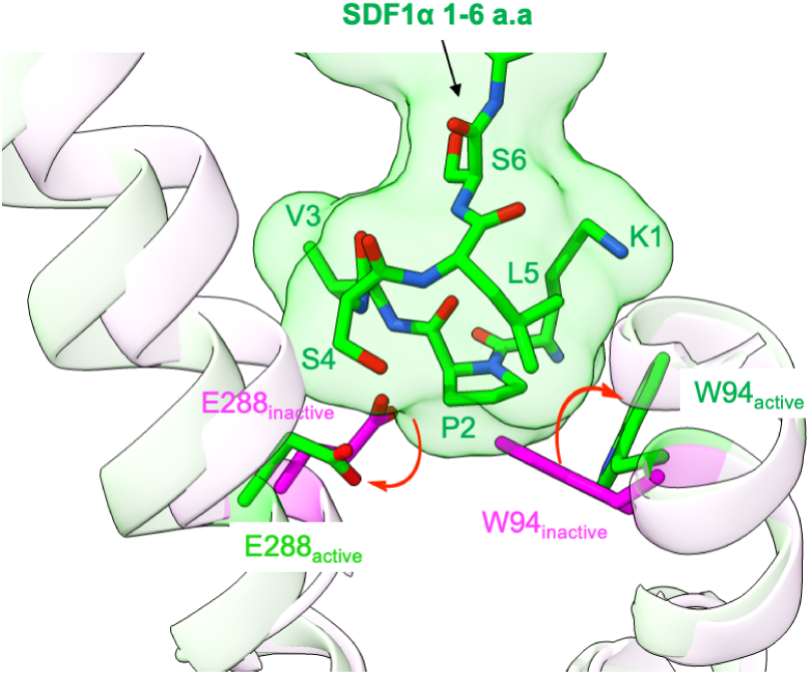
Conformational changes of W94 and E288 triggered by the N-terminal residues of SDF-1α. The inactive CXCR4 structure is shown in pink (PDB ID: 3ODU), and the active CXCR4 structure is shown in green (PDB ID: 8U4O).

Using these structural insights, we employed targeted molecular dynamics (TMD) simulations^31,32^ to model the activation process of CXCR4, capturing intermediate conformations along the activation pathway. Coarse-grained (CG) energy calculations further revealed the free-energy landscape of this activation process. An overview of the computational workflow employed in this study is provided in Figure S2. The results highlight three major energy barriers. The first transition state (TS1), associated with the movement of helices TM5 and TM6, acts as the rate-limiting step. The second transition state (TS2), involving conformational changes in the active-site residue E288, presents the lowest energy barrier. The third transition state (TS3) corresponds to the rearrangement of multiple conserved residues critical for GPCR function, including W94, W252, and F248. The specific active conformations of E288 and W94 within the CXCR4 active pocket were found to reduce the activation energy barriers of TS2 and TS3. Furthermore, by alanine scanning, we identified critical residues within the ligand-binding and cholesterol-binding pockets that play pivotal roles in these barriers. These findings reveal that CXCR4 activation is governed by the coupling of multiple functional sites, each contributing to the receptor’s conformational transitions.

Based on insights from our computational studies, we successfully developed a small-molecule CXCR4 agonist, designated as HL82624. Through a dual-moiety design strategy previously used in the design of the CXCR4 peptide agonist SDV1a,^24^ we incorporated the first two residues of the SDF-1α N-terminus, Lysine and Proline, as the signaling moiety to stabilize the active conformation of W94 and E288 that could reduce the CXCR4 activation energy barrier, while the binding moiety derived from our CXCR4 antagonist HF51116 ^13,33^ acted to provide additional receptor interaction. HL82624 displayed CXCR4 binding and activation activities measured by the enhanced CXCR4-mediated cell migration. These results for the designed agonist, together with computational results of CXCR4 activation mechanism analysis described above, demonstrate a theoretical framework for understanding the activation mechanism of CXCR4 and lay an experimental foundation for the development of next-generation agonists with enhanced binding affinity and therapeutic potential.

## Results and Discussions

### CG Free Energy Profile for CXCR4 Activation

To investigate the conformational free energy landscape of CXCR4 activation, we constructed models based on existing cryo-EM structures obtained from the Protein Data Bank (PDB). Specifically, the CXCR4 inactive state induced by IT1t (PDB ID: 3ODU^34^) and the active states induced by SDF-1α and SDV1a (PDB IDs: 8U4O^35^ and 9UPU^24^) were selected as endpoints to generate intermediate geometries and, thus, the conformational landscape of CXCR4 activation by Targeted Molecular Dynamics (TMD) simulations. Thirty intermediate structures were uniformly extracted for each activation process to assemble its corresponding conformational free energy profile, as depicted in Figure 2A and B. These profiles describe the transition of CXCR4 from the IT1t-induced inactive state (PDB ID: 3ODU) to the agonist-induced active states of SDF-1α (PDB ID: 8U4O) and SDV1a (PDB ID: 9UPU). Both activation processes revealed three distinct transition states (TS1, TS2, and TS3), with TS1 identified as the rate-limiting step. The energy barrier for TS1 was 12.46 kcal/mol for SDF-1α-induced activation and 8.67 kcal/mol for SDV1a-induced activation.

**Figure 2:**
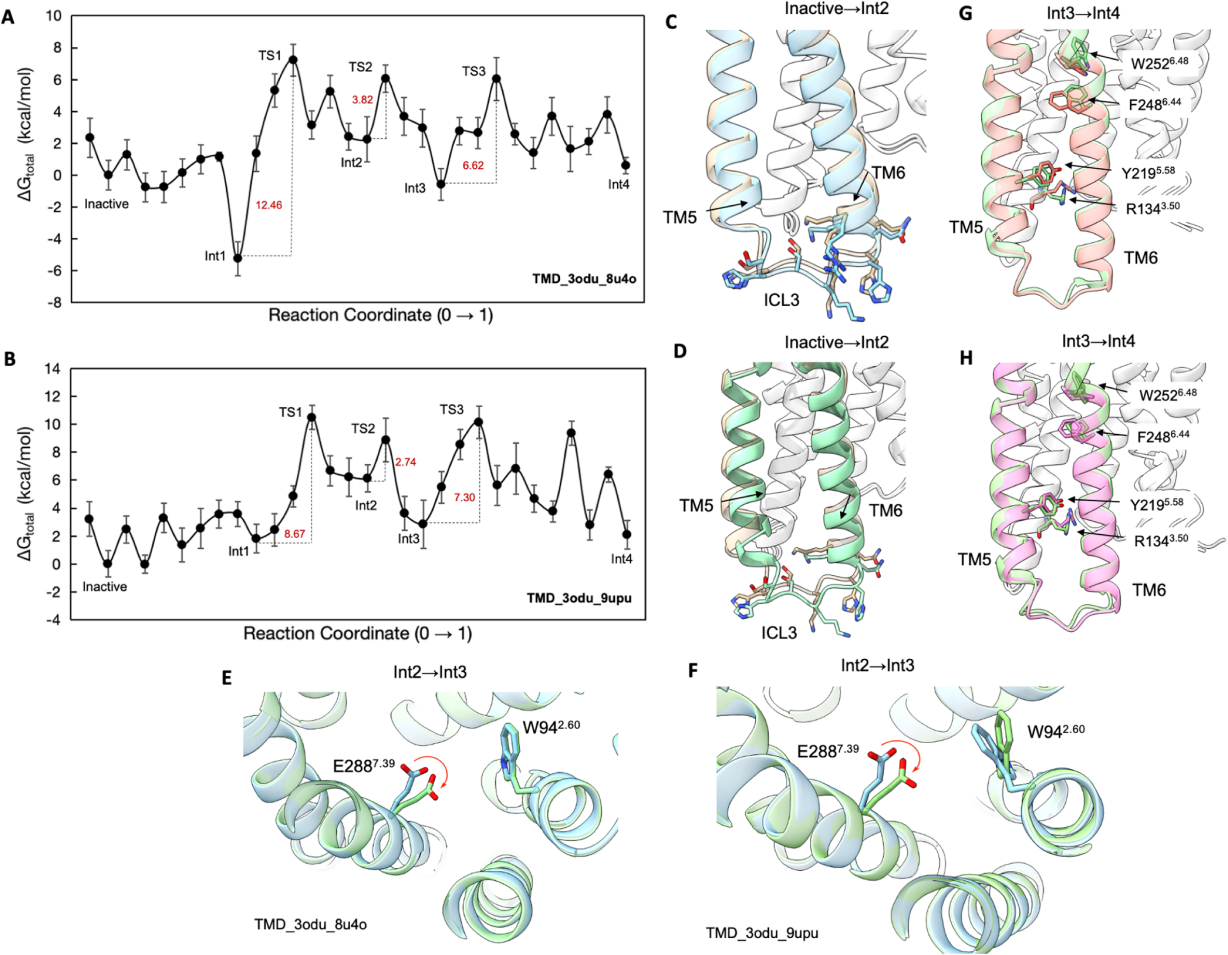
CG conformational free energy profiles of CXCR4 activation and observed conformational changes along the activation. (A) Free energy profile for the activation process between the IT1t-induced inactive state (PDB ID: 3ODU) and the SDF-1α-induced active state (PDB ID: 8U4O). The *x*-axis represents the conformational change describing the activation pathway of CXCR4 from the inactive to the active state, whereas the *y*-axis corresponds to the total free energy of the system relative to the inactive state along this pathway. (B) Free energy profile for the activation process between the IT1t-induced inactive state (PDB ID: 3ODU) and the SDV1a-induced active state (PDB ID: 9UPU). (C,D) Conformational changes during the conversion from the inactive state to the Int2 one (C: TMD_3odu_8u4o, D: TMD_3odu_9upu). (E,F) Conformational changes during the conversion from the Int2 state to the Int3 one (E: TMD_3odu_8u4o, F: TMD_3odu_9upu). (G,H) Conformational changes during the conversion from the Int3 state to the Int4 one (G: TMD_3odu_8u4o, H: TMD_3odu_9upu).

SDV1a, a CXCR4 peptide agonist previously developed in our laboratory by using a dual-moiety strategy, incorporates the first eight residues of SDF-1α to elicit agonistic activity, while the last 21 residues are derived from the CXCR4 antagonist VMIP-II.^23,24^ This design results in slightly weaker agonistic effects compared to SDF-1α, which is consistent with the relatively weaker conformational changes induced by SDV1a compared to the native ligand,^24^ despite the slightly lower energy barrier observed for TS1 in the SDV1a-induced activation pathway. TS2 exhibited the lowest energy barriers in both systems, with values of 3.82 kcal/mol for SDF-1α-induced activation and 2.74 kcal/mol for SDV1a-induced activation. By contrast, TS3 displayed higher energy barriers of 6.62 and 7.30 kcal/mol for SDF-1α-induced and SDV1a-induced activation, respectively.

Structural analysis provides further insights into the mechanisms behind CXCR4 activation. TS1 was found to involve structural perturbations primarily in the intracellular loop 3 (ICL3) and movements of transmembrane helices TM5 and TM6 (Figure 2C and D). This observation indicates that the rate-limiting step of CXCR4 activation is not associated with conformational changes in the extracellular ligand-binding pocket but is instead highly dependent on structural rearrangements near the intracellular regions. This finding aligns with previous studies suggesting that CXCR4 activation is initiated by G protein precoupling.^28,29^ TS2 was associated with conformational changes of the active-site residue E288, implying that its energy barrier arises from the rearrangement of the E288 side chain (Figure 2E and F). Notably, in the MD trajectory, we observed that significant structural variation of W252 follows the conformational change of E288. This suggests a signaling linkage between the residue E288 in the active pocket and critical downstream residues (Supplementary movie). TS3 involved conformational rearrangements of multiple conserved residues critical for GPCR function, including W252^6.48^, F248^6.44^, Y219^5.58^, and R134^3.50^ (Figure 2G and H). These residues are distributed across the transmembrane regions of CXCR4, indicating that TS3’s energy barrier is driven by multisite conformational changes.

### Specific Side-Chain Conformation of W94 and E288 for CXCR4 Activation

Recently, we utilized cryo-EM to resolve the structures of CXCR4 in antagonist-bound states with AMD3100, AMD070, and HF51116 ^30^ as well as in active states bound to the synthetic agonists SDV1a and SDVX1 coupled with G proteins.^24^ By comparing the antagonist-bound and agonist-bound CXCR4 structures, we identified significant conformational differences in two key residues within the active site of CXCR4, W94 and E288 (Figure 1). Notably, in the active-state structures, W94 adopts a vertical conformation, in contrast to the flattened conformation observed in the antagonist-bound states. These structural differences prompted further investigation into the roles of W94 and E288 in CXCR4 activation.

To evaluate the contributions of these residues to CXCR4 activation, we constructed two models for the analysis. In the first model, the side chain of W94 in the inactive structure (PDB ID: 3ODU) was modified from the antagonist-like flattened conformation to the agonist-like vertical conformation. In the second model, both the W94 and E288 side chains were adjusted to their active-state conformations. Targeted MD and CG calculations were then performed to determine the free energy profiles, as shown in Figure 3A and B. The results reveal distinct impacts of these conformational changes on the transition state energy barriers. In Figure 3A, modifying W94 to its active-state conformation led to a substantial reduction in the energy barrier of TS3, while TS1 (12.46 to 8.44 kcal/mol) and TS2 (3.82 to 3.77 kcal/mol) remained largely unaffected. In Figure 3B, adjusting both W94 and E288 to their active-state conformations resulted in the disappearance of the TS2 barrier and a slight reduction in the TS1 (12.46 to 9.13 kcal/mol) and TS3 barriers (6.62 to 4.53 kcal/mol). These findings suggest that the conformational rearrangement of W94 predominantly influences the energy barrier at TS3, whereas the rearrangement of E288 primarily affects TS2. This observation is consistent with our earlier conclusions that TS2 is driven by the side-chain rearrangement of E288 (Figure 2E and F). Overall, these results highlight the pivotal roles of W94 and E288 in CXCR4 activation. The distinct contributions of these residues to specific transition states provide valuable insights at the molecular level into the CXCR4 activation mechanism and suggest potential strategies for the rational design of targeted agonists.

**Figure 3:**
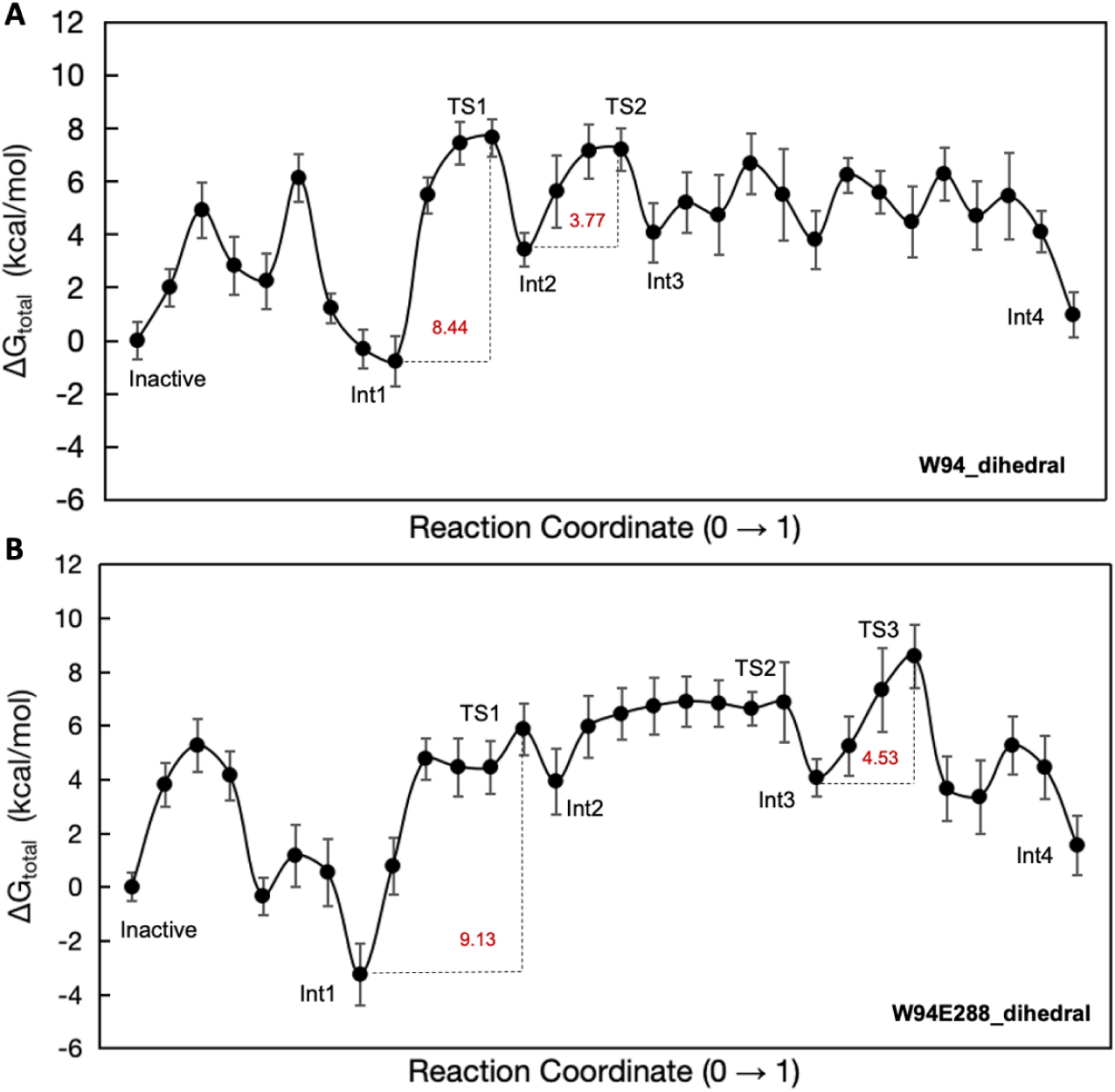
Effects of the W94 and E288 side chain conformations on the CXCR4 conformational free energy profiles for the activation process between the IT1t-induced inactive state (PDB ID: 3ODU) and the SDF-1α-induced active state (PDB ID: 8U4O). (A) W94 was modified to its active conformation. (B) Both W94 and E288 were modified to their active conformations.

### Partial Agonist AMD3100 Binding for CXCR4 Activation

To further investigate the role of ligands in CXCR4 activation, we study the AMD3100-induced CXCR4 inactive and active states (PDB IDs 8ZPN^30^ and 8U4P,^35^ respectively). Biochemical experiments have previously demonstrated that AMD3100 acts as a partial agonist, exhibiting a weak ability to promote cell migration.^36^ Analysis of the cryo-EM structures for such states revealed that AMD3100 stabilizes the active conformations of key residues W94, E288, and W252 within CXCR4 but fails to induce the downstream rearrangement of the PIF motif and helices’ movement.^24^ To better understand the effects of such ligand, the conformational free energy profile for the CXCR4 activation process governed by AMD3100 binding was calculated, as shown in Figure 4A. The results reveal a significant change in the free energy landscape. Unlike the conformational free-energy profiles seen so far (Figure 2), the AMD3100-induced system exhibited a single transition state with a markedly reduced energy barrier of 5.56 kcal/mol. These findings indicate that AMD3100 can significantly decrease the activation energy barrier of CXCR4.

**Figure 4:**
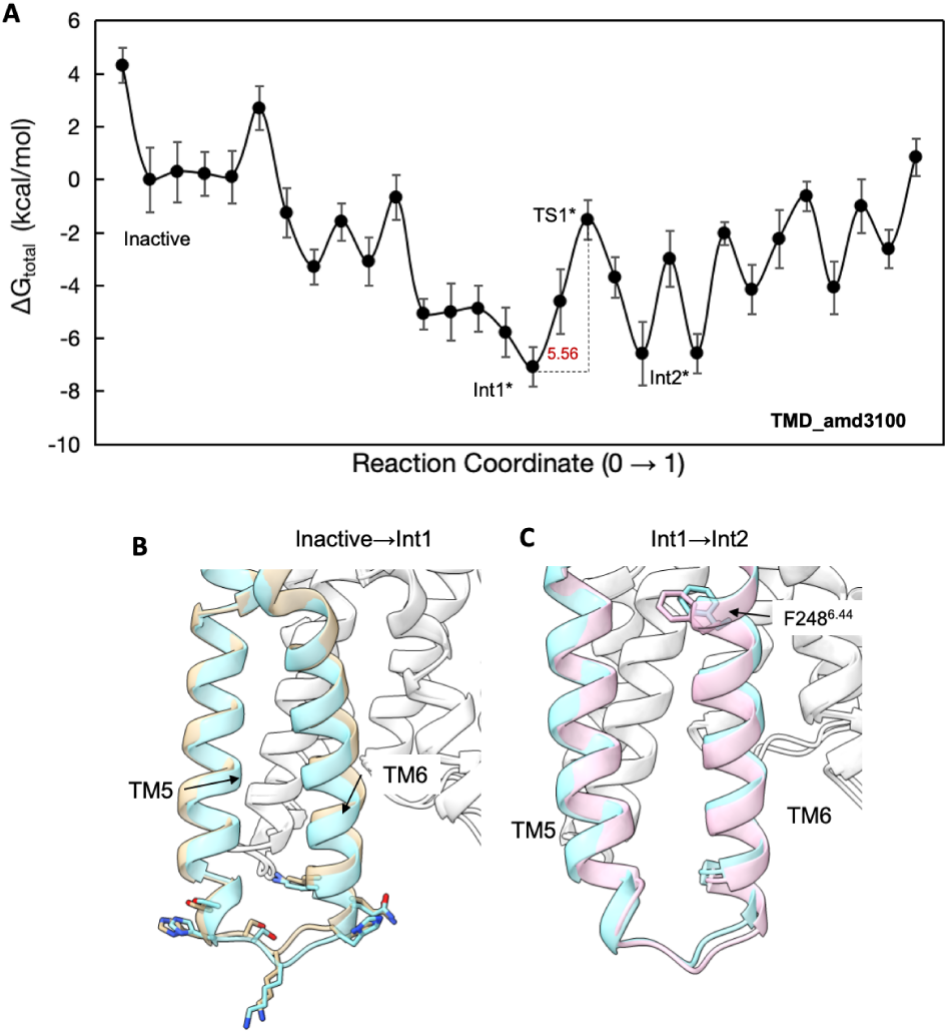
(A) Free energy profile for the CXCR4 activation process between the AMD3100-induced inactive and active states (PDB IDs: 8ZPN and 8U4P, respectively). (B) Conformational changes during the conversion from the inactive state to the Int1 one. (C) Conformational changes during the conversion from the Int1 state to the Int2 one.

Structural analysis provides further insights into the underlying mechanisms. AMD3100 was found to stabilize intermediate state 1, promoting structural rearrangements in intracellular loop 3 (ICL3) and facilitating the movement of transmembrane helices TM5 and TM6 (Figure 4B). The transition state in the AMD3100-bound system was associated with the rearrangement of residue F248 (Figure 4C), a critical component of the PIF motif. As a connector residue essential for GPCR activation, F248 rearrangement constitutes a key step in the activation process.^37–39^ However, AMD3100 did not significantly lower the energy barrier associated with the PIF motif rearrangement. These findings underscore the role of ligand binding in CXCR4 activation: lowering the TS1 energy barrier for G-protein coupling.

### Mutational Effects of Key Residues for CXCR4 Activation

To systematically investigate the contributions of individual residues to CXCR4 activation, particularly those located around the ligand- or cholesterol-binding sites, free energy profiles were calculated for the activation process of 68 CXCR4 variants derived from alanine scanning (Tables S1 and S2). These residues were selected based on prior biochemical studies suggesting their involvement in receptor activation,^27^ as well as structural differences observed between resolved inactive and active CXCR4 structures. For each mutant system, we conducted a Targeted MD simulation and CG conformational free energy calculations to determine the effects of the considered mutation on the activation energy barrier. The change in the energy barrier for each mutation was quantified as ΔΔG^‡^ = ΔG^‡^_mutant_ – ΔG^‡^_WT_. A positive ΔΔG^‡^ (ΔΔG^‡^ > 0) indicates an increased activation energy barrier, thereby inhibiting CXCR4 activation, while a negative ΔΔG^‡^ (ΔΔG^‡^ < 0) suggests a decreased activation energy barrier, facilitating receptor activation. The mutational effects on TS1 and TS3 are depicted in Figure 5A and B, respectively.

**Figure 5:**
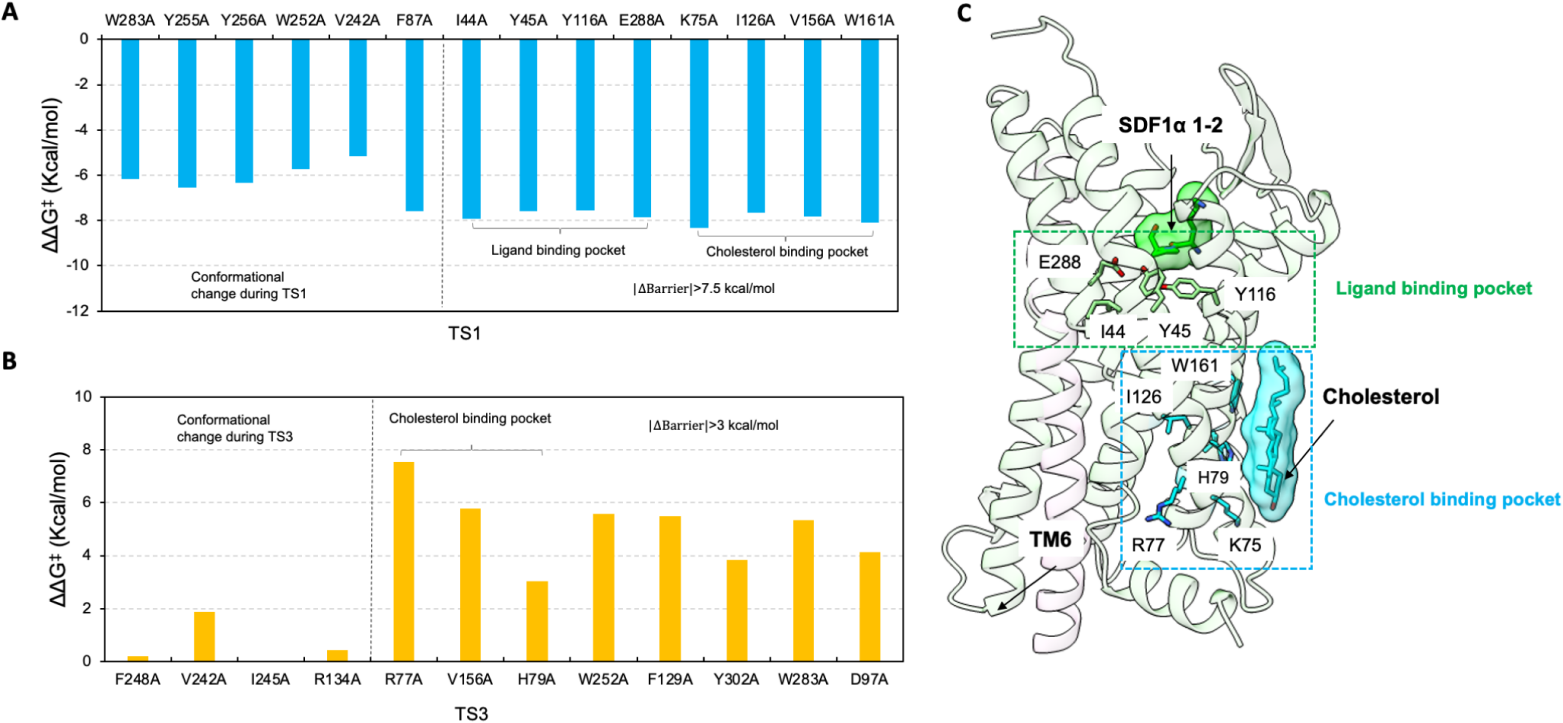
Mutational effects on the energy barrier of TS1 (A) and TS3 (B). (C) The ligand binding pocket and cholesterol binding pocket.

Interestingly, we observed opposing mutational effects on TS1 and TS3, revealing a crosstalk between these two transition states (Table S2). For TS1, mutations predominantly facilitated activation (ΔΔG^‡^ < 0), suggesting that the wild-type residues might initially hinder the activation event. Conversely, for TS3, mutations predominantly inhibited activation (ΔΔG^‡^ > 0), implying that the wild-type residues play a crucial role in promoting this last step of the activation process. The mutational results for TS1 are shown in Figure 5A. Mutations to alanine of residues that undergo conformational changes during this transition state, such as Y255^6.51^, Y256^6.52^, W252^6.48^, and V242^6.38^, significantly reduced the TS1 energy barrier. This indicates their direct involvement in helix formation during this transition state. Notably, residues in the ligand-binding pocket (e.g., I44^1.38^, Y45^1.39^, and Y116^3.32^) and the conserved cholesterol-binding pocket (e.g., K75^2.41^, I126^3.42^, V156^4.45^, and W161^4.50^) (Figure 5C) exhibited the most pronounced effects on TS1, with ΔΔG^‡^ values dropping below −7.5 kcal/mol. These findings suggest that these residues play critical roles in stabilizing the receptor in its inactive state, contributing substantially to the TS1 energy barrier and shaping the energy landscape of early activation.

In contrast, the mutational results for TS3, shown in Figure 5B, reveal a different pattern. Residues which undergo conformational changes during TS3, such as F248^6.44^, V242^6.38^, I245^6.41^, and R134^3.50^, showed minimal changes to the TS3 barrier when mutated to alanine. This suggests that while these residues undergo structural rearrangements during the activation process, they are not the primary contributors to the TS3 energy barrier. These results imply that PIF motif rearrangement, observed during TS3, may represent a passive process occurring in the later stages of receptor activation. Instead, residues with the largest ΔΔG^‡^ values, including those in the conserved cholesterol-binding pocket (e.g., R77^2.43^, V156^4.45^, and H79^2.45^), the G protein-binding region (e.g., F129^3.45^ and Y302^7.53^), and the CWXP motif (e.g., W252^6.48^), collectively appear to play pivotal roles in promoting receptor activation.

Overall, our alanine scanning analysis indicates that CXCR4 activation is likely a multisite cooperative process, where residues contribute in both direct and indirect ways. While many residues do not participate directly in the conformational changes associated with activation, their presence seems to support and drive the activation process. Notably, our findings highlight the critical role of cholesterol in both the early and late stages of CXCR4 activation. These results align with previous experimental evidence suggesting that cholesterol is essential for CXCR4’s physiological function, further emphasizing the importance of this lipid in modulating receptor activity.^40,41^ Of particular interest is the emerging pattern that residues seem to adopt distinct roles at different stages of activation. During early activation, many residues appear to act as inhibitors, stabilizing CXCR4 in its inactive state and preventing premature activation. However, in the later stages of activation, residues appear to act as agonists, actively promoting receptor activation. This difference underscores the dynamic nature of CXCR4 activation and highlights the complex interplay of residue-specific contributions throughout the process.

### Rational Design of Small-Molecule CXCR4 Agonists

In this study, we first observed significant conformational changes in the side chains of W94 and E288 within the receptor’s orthosteric site upon the binding of agonist peptides by comparing recently resolved CXCR4 antagonist-bound and agonist-bound structures. This initial observation suggests that these two residues may act as signal-initiating switches within the active site. Our computational investigations further revealed that stabilizing the active side-chain conformations of W94 and E288 significantly lowers the activation energy barriers of CXCR4. These findings highlight that the conformational changes of these two residues are driven by agonist peptide binding, particularly the flipping motion of the W94 side chain, rather than being random or inconsequential side-chain perturbations. These results underscore the functional importance of W94 and E288 in CXCR4 activation and provide mechanistic insights into their roles as critical molecular switches in the receptor’s activation pathway.

Based on this, we hypothesized that stabilizing these residues in their active conformations could be a viable strategy for developing effective small-molecule CXCR4 agonists. To test this hypothesis, we focused on the two N-terminal residues of SDF-1α, Lys and Pro, which directly interact with W94 and E288 and have been previously identified as essential for the agonistic activity of SDF-1α.^22,42,43^ The binding mode of HF51116, a CXCR4 antagonist we previously developed,^13,30,33^ revealed that it occupies both the minor pocket and the major pocket of CXCR4 (Figure 6A). Notably, the [3-(cyclohexylamino)­propyl]­amino fragment (**F01**) of HF51116, which occupies the minor pocket, overlaps with the pocket occupied by Lys–Pro in SDF-1α. Based on this observation, we retained the HF51116 fragment that binds to the major pocket and replaced its **F01** fragment with Lys–Pro, resulting in the design of the novel compound HL82624 (Figure 6B).

**Figure 6:**
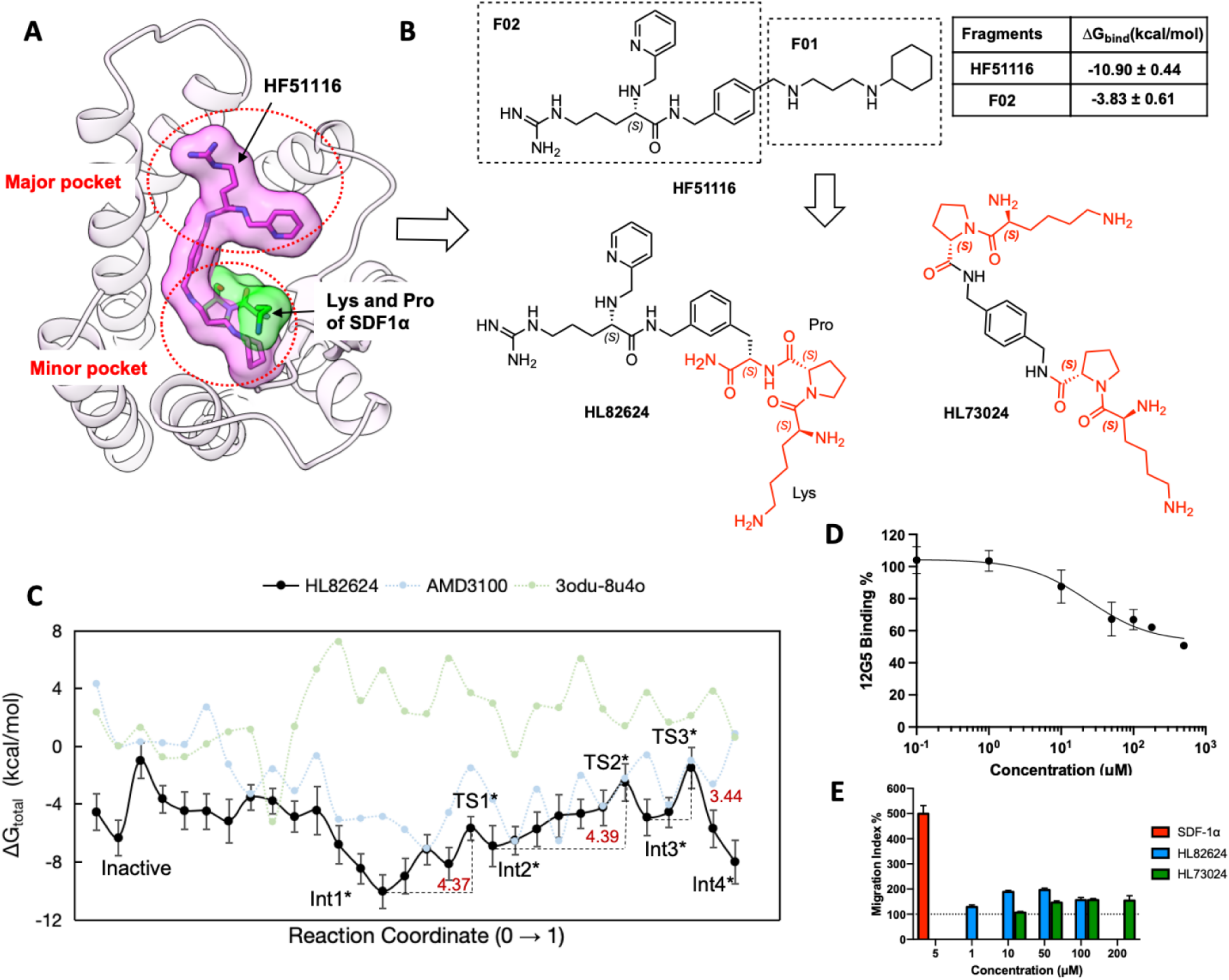
Rational design of small-molecule CXCR4 agonists. (A) Overlap of HF51116 and Lys–Pro in the minor pocket of CXCR4. (B) Binding free energy decomposition analysis of HF51116, and chemical structures of HL82624 and HL73024. (C) Free energy profile for the activation process between the HL82624-induced inactive state (averaged from two MD poses) and the SDF-1α-induced active state (PDB ID: 8U4O). (D) Competitive binding activity of HL82624. (E) Cell migration-promoting effects of HL82624 and HL73024. SDF-1α (5 nM) was used as a positive control. The migration index of 100% was the background value. All experimental data were expressed as means ± SEM from at least three independent experiments.

To assess whether HL82624 exhibits agonistic activity, we calculated the CXCR4 activation energy barrier using the above-built TMD-CG approach. The initial HL82624-bound CXCR4 conformation was generated using constrained induced-fit docking with Maestro to preserve the original interactions of its constituent fragments, explicitly accounting for the flexibility of W94. Subsequently, three independent MD simulations of 500 ns were carried out with positional restraints applied to HL82624. Frames corresponding to 200 and 500 ns were selected to capture representative stabilized and post-MD conformations, which served as initial states for TMD-CG simulations toward the SDF-1α-induced active conformation (PDB ID: 8U4O).

The resulting conformational energy profile reveals that HL82624 substantially reduces the energy barrier associated with the first transition state of CXCR4 activation (Figure 6C). The averaged CG curves derived from the two HL82624-induced initial states closely resemble those obtained for the AMD3100-CXCR4 system. This similarity suggests that HL82624, like AMD3100, may function as a partial agonist by facilitating early-stage CXCR4 activation. Furthermore, binding free energy decomposition analysis of HF51116 revealed that its **F02** fragment, which occupies the major pocket, contributes favorably to binding affinity (−3.83 kcal/mol, Figure 6B). The benchmark results of the binding free energy calculations are presented in Table S3 and Figure S3. These TMD-CG and binding energy calculations provide a rational basis for the design of HL82624 and support our hypothesis that replacing the minor pocket-binding fragment with Lys–Pro can convert the antagonist HF51116 into a small-molecule agonist. Following the design and computational validation, we synthesized HL82624 and tested its biological activities. CXCR4 competitive binding activities were measured using Sup-T1 cells and anti-CXCR4 monoclonal antibody 12G5. HL82624 could effectively bound to Sup-T1 cells with an IC_50_ of 24 μM (Figure 6D). The cell migration results indicate HL82624 effectively promotes cell migration within the micromolar range, exhibiting maximal activity at 10–50 μM (Figure 6E). These results validate our design strategy and highlight the utility of the TMD-CG approach for assessing the agonistic potential of novel small-molecule ligands.

We also evaluated several literature-reported small-molecule CXCR4 agonists (NUCC-390,^44^ compound 1,^45^ and compound 6a^46^); however, under our assay conditions, we were unable to detect any CXCR4 binding activity of none of them in the 12G5 competitive binding assay. Taken together, these results suggest that HL82624 may represent the first small-molecule agonist with direct evidence of binding to the orthosteric pocket of CXCR4.

Finally, to further examine whether the two N-terminal residues of SDF-1α, Lys and Pro, are sufficient to confer agonistic activity, we designed and synthesized HL73024, a symmetrical molecule in which a benzene ring serves as the central scaffold and is para-disubstituted with two identical Lys–Pro moieties. As shown in Figure 6D, HL73024 promoted cell migration in chemotaxis assays, exhibiting CXCR4-mediated migratory activity at concentrations ranging from 50 to 200 μM. These results indicate that the Lys–Pro structural motif constitutes a minimal functional unit capable of activating CXCR4. On the other hand, HL73024 lacks the CXCR4 major pocket–binding motif derived from HF51116 compared with HL82624, which weakens its interactions within the binding pocket. The binding modes of HL73024 and HL82624 are shown in Figure S4 for comparison. HL73024’s agonist effect is weaker than that of HL82624 which is consistent with the design notion that both Lys–Pro and HF51116 motifs are necessary for better CXCR4 activation activity.

## Conclusion

This study advances our understanding of CXCR4 activation through an integrated computational and experimental framework. Our computational analyses reveal W94 and E288 as critical residues that serve as a molecular switch in the active site of CXCR4 for receptor activation. Based on these mechanistic insights, we successfully designed and developed a novel small-molecule agonist, HL82624, which demonstrated promising binding affinities and functional activity in promoting cell migration. The success of HL82624 further validates the effectiveness of the dual-moiety strategy that we previously employed in the development of CXCR4 peptide agonists. In this strategy, the binding motif is responsible for providing high binding affinity, while the signaling motif is essential for activating CXCR4. In earlier peptide agonist designs, the first eight amino acids of SDF-1α were commonly used as the signaling motif. However, in this study, through developing a symmetrical molecule HL73024, we demonstrated that retaining only the first two amino acids of SDF-1α was sufficient to achieve receptor activation

Alanine scanning highlighted the dynamic roles of residues, transitioning from stabilizing the inactive state to facilitating activation in the later stages. Furthermore, our analyses revealed that the conserved cholesterol-binding pocket plays a pivotal role in CXCR4 activation. Cholesterol appears to stabilize key intermediate states and contributes to both the early and late stages of receptor activation, aligning with previous findings that cholesterol is essential for CXCR4’s physiological function.

For future development of CXCR4 small-molecule agonists, fragments capable of stabilizing the active conformations of W94 and E288 within the minor pocket might serve as prosignaling elements that facilitate receptor activation. When combined with moieties that interact with the major pocket to augment the binding affinity, such hybrid molecules may achieve both binding and activation capabilities. The design and computational characterization of HL82624 illustrate the feasibility of this strategy. More broadly, the distributed nature of the activation barrier identified in this work highlights the intrinsic challenge of designing small-molecule CXCR4 agonists. Future efforts may benefit from ligands capable of engaging broader receptor surfaces or targeting regions more directly coupled to intracellular activation pathways. Overall, this study provides mechanistic insights into the energetic and structural determinants of CXCR4 activation and establishes a rational framework for the development of CXCR4 agonists with potential therapeutic applications in neurodegenerative diseases and regenerative medicine.

## Supplementary Material





## Data Availability

The dataset underlying this work is available free of charge at https://dataverse.csuc.cat/dataverse/UPC with a persistent DOI: https://doi.org/10.34810/data3229. The dataset contains input files, starting structures and developed specific parameters necessary to reproduce calculations of the present paper.

## Notes

Computational details, 12G5 competitive binding assay, Cell migration assay, synthetic route for compounds HL82624 and HL73024, synthetic experimental details and NMR spectrums of HL82624 and HL73024 (PDF)
Signaling linkage movie between the residue E288 and W252 (MOV)
