# Supplementary material for: Computational Insights into the Activation Mechanism of CXCR4: Implications for the Design of Small Molecule Agonists
Source: J Am Chem Soc. 2026 May 13;148(20):20545–54. doi: 10.1021/jacs.6c01087 (PMC13220262; doi:10.1021/jacs.6c01087)
Supplement: Supplementary file 1 [file ja6c01087_si_001.pdf]

# Computational Insights into the Activation Mechanism of CXCR4: Implication for the Design of Small Molecule Agonists

Jiao Zhou[a,b,#], Xiang Liu[a,c,#], Yan Xu[c,#], Alejandro Cruz[d,e,*], Jing An[c,*], Arieh Warshel[e,*], Ziwei Huang[c,*]

[a]School of Chemistry and Chemical Engineering, Guangdong Pharmaceutical University, Zhongshan, 528458, China

[b]Ciechanover Institute of Precision and Regenerative Medicine, School of Medicine, Chinese University of Hong Kong, Shenzhen, 518172, China

[c]Department of Medicine, Division of Infectious Diseases and Global Public Health, School of Medicine, University of California at San Diego, La Jolla, California, 92037, USA

[d]Departament d'Enginyeria Química (EQ), ETSEIB, Universitat Politècnica de Catalunya - BarcelonaTech (UPC), Campus Sud, Edif. PG, Av. Diagonal, 647, 08028 Barcelona, Spain

[e]Department of Chemistry, University of Southern California, Los Angeles, California, 90089, USA

[#]These authors contributed equally: Jiao Zhou; Xiang Liu; Yan Xu

[*]Corresponding authors: Alejandro Cruz (alejandro.cruz.saez@upc.edu); Jing An (jan@health.ucsd.edu); Arieh Warshel (warshel@usc.edu); Ziwei Huang (zhuang@health.ucsd.edu)

## Table of Contents

I. Cholesterol binding observed in the active-state structures of CXCR4……...S2

II. Methods……………………………………………………………………S3

Computations details………………………………………………………S3

Biological assay details……….………………………………………...S14

Synthetic details…………………………………………………………...S15

III. $^{1}$H NMR, $^{13}$C NMR, $^{19}$F NMR spectra of target compounds………………...S20

IV. Supplementary references…………………………………………..….…S22

# I. CHOLESTEROL BINDING OBSERVED IN THE ACTIVE-STATE STRUCTURES OF CXCR4

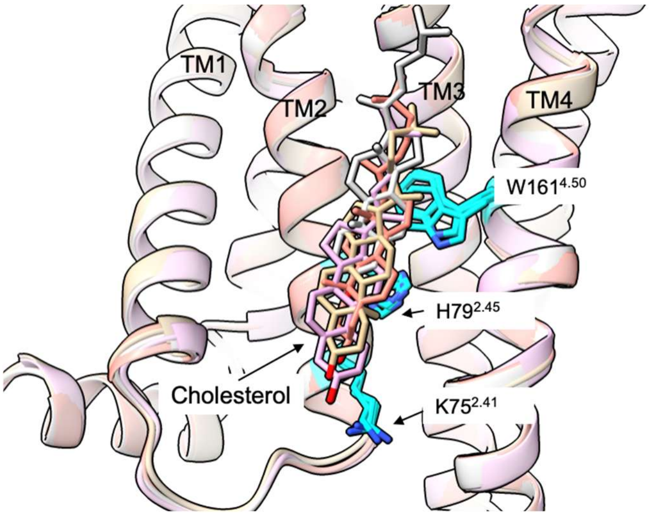


**Figure S1.** Cholesterol binding observed in the active-state structures of CXCR4. (PDB IDs: 8U4O, 8U4P, 9UPU, and 9UPV).

## II. METHODS

### Computational details

In this work, a series of computational approaches were employed to investigate the activation mechanism of CXCR4 and to support the rational design of small-molecule CXCR4 agonists. For clarity, **Figure S2** provides a flowchart summarizing the computational workflow employed in this study. Unless otherwise specified, conformational free energy calculations and binding free energy calculations were performed using the Molaris-XG package[1], while conventional all-atom MD simulations were carried out using the GROMACS 2021 package[2]. Structural visualization and molecular representations were generated using UCSF Chimera[3] or PyMOL (Version 2.0 Schrödinger, LLC.).

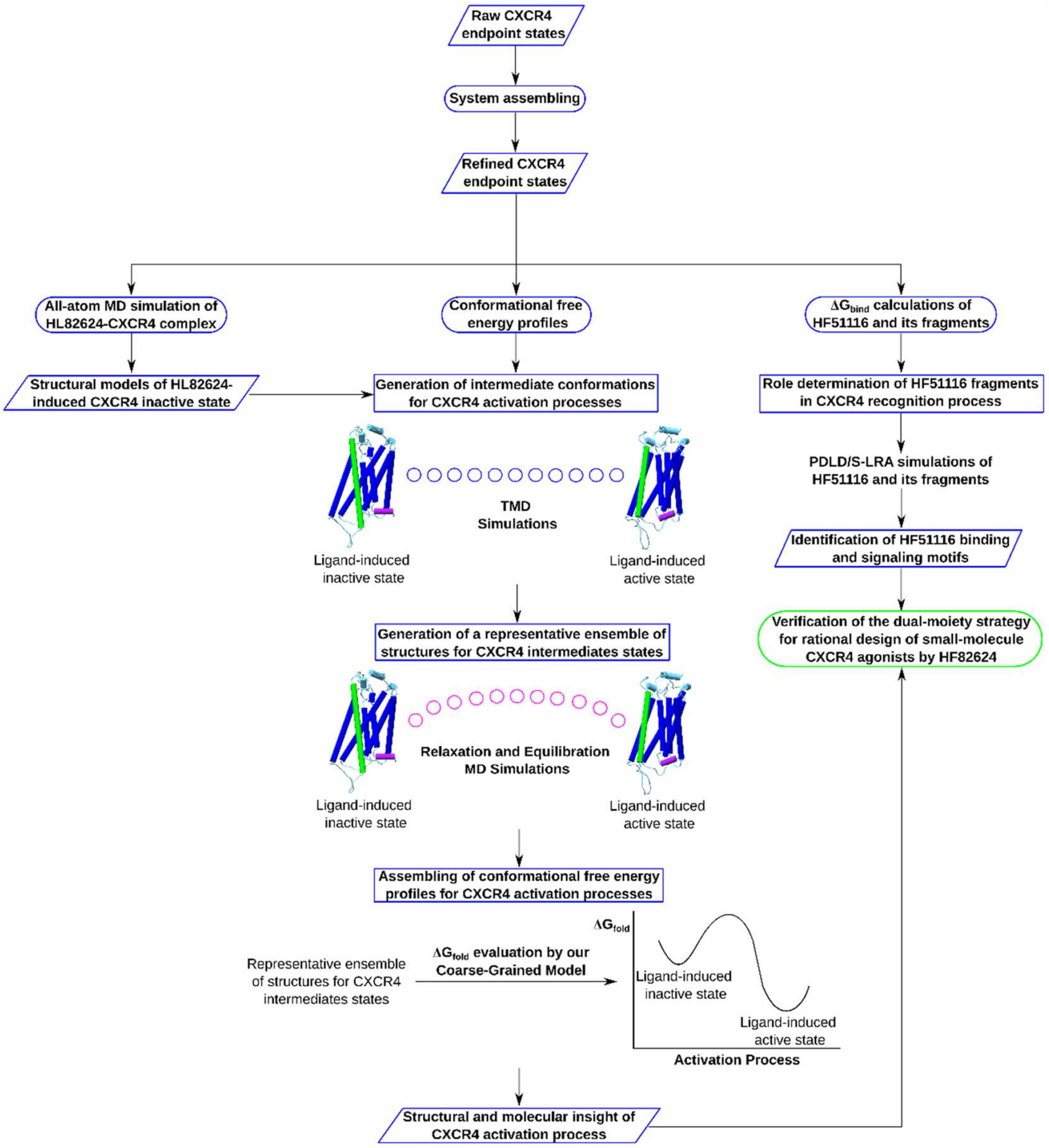


**Figure S2.** Computational workflow for investigating the activation mechanism of CXCR4.

In this workflow, the left branch illustrates the all-atom MD simulation, the central branch depicts conformational free-energy calculations, and the right branch shows binding free-energy calculations. **First**, representative ensembles of CXCR4 endpoint states (inactive and actives) were selected to explore the conformational landscape of CXCR4 activation. Refined structural models were obtained either through a unified processing protocol applied to the raw structures or through a conventional all-atom MD simulation (left branch). **Second**, based on these refined models, conformational free-energy ($\Delta G_{fold}$) profiles describing CXCR4 activation were calculated for multiple activation processes (central branch). Intermediate conformations required for constructing these profiles were generated using targeted MD simulations, while representative structural ensembles for each intermediate state were obtained through relaxation and equilibration MD simulations. The $\Delta G_{fold}$ values were subsequently estimated using a refined coarse-grained model for membrane proteins. **Third**, binding free-energy calculations ($\Delta G_{bind}$) were performed using PDLD/S-LRA simulations to evaluate the contributions of HF51116 fragments to CXCR4 recognition (right branch). Insights obtained from the $\Delta G_{fold}$ profiles and $\Delta G_{bind}$ calculations guided the proposal of a dual-moiety strategy for the rational design of small-molecule CXCR4 agonists, which was validated by the design of HL82624 (highlighted in green).

## 1. System assembly

The initial structures used in all calculations and simulations presented in this work were derived from the following CXCR4 states (active and inactive):(1) IT1t-induced inactive state (PDB ID: 3ODU); (2) SDF-1α-induced active state (PDB ID: 8U4O); (3) SDV1a-induced active state (PDB ID: 9UPU); (4) AMD3100-induced inactive state (PDB ID: 8ZPN); (5) AMD3100-induced active state (PDB ID: 8U4P); (6) HF51116-bound inactive state (PDB ID: 8ZPL).

A unified protocol was applied to generate refined CXCR4 endpoint states from the corresponding experimental structures. First, the structures were cleaned by removing G proteins, crystallization agents, and orthosteric ligands (for ligand-induced receptor only). The sequence in each PDB structure was then aligned with the wild-type human CXCR4 sequence (UniProt ID: P61073; CXCR4_HUMAN) to revert mutations, insertions, and engineered substitutions present in the crystal or cryo-EM structures, as well as to reconstruct missing residues. Following sequence restoration, local structural refinements were performed through energy minimization to relieve steric clashes introduced during the back-mutation process. All structure editing, sequence restoration, and energy minimization procedures were carried out using the Schrödinger Maestro software package. After obtaining the processed CXCR4 endpoint structures,

protonation states were assigned at pH 7.0 using the Monte Carlo Proton Transfer (MCPT) method[4] implemented in the Molaris-XG package. A total of $10^5$ MCPT steps were performed to ensure convergence of the electrostatic free energy of the folded protein. Finally, disulfide bridges were introduced between the cysteine pairs Cys28–Cys274 and Cys109–Cys186 to generate the final refined CXCR4 endpoint states used in subsequent calculations.

## 2. Conformational free energy profiles

The conformational landscape of CXCR4 activation was explored using a total of 75 distinct activation systems (**Table S1**), derived from both wild-type and modified CXCR4 endpoint states. Regarding modified CXCR4 endpoint states, 68 alanine variants were generated using the PyMOL mutagenesis tool (**Table S2**), with residues selected based on their putative roles in CXCR4 activation, as well as structural differences observed between resolved inactive and active CXCR4 structures. In addition, active-state rotamers of W94, and of W94 together with E288, were manually adjusted in the IT1t-bound CXCR4 inactive state to adopt the conformations observed in the SDF-1α-bound active state, representing a fully activated CXCR4 conformation. These profiles were assembled by combining three types of simulations (see **Figure S2**): (1) Targeted molecular dynamics (TMD) simulations to generate intermediate conformations along the considered activation pathways; (2) Relaxation and equilibration MD simulations to obtain representative structural ensembles for these intermediate states; and (3) Coarse-grained (CG) calculations to estimate the CXCR4 conformational free energy ($\Delta G_{fold}$) of the previously generated structural ensembles.

**Table S1. 75 CXCR4 activation systems used in this study**

| Number | Inactive state | Active State | Modifications |
|---|---|---|---|
| 1 | IT1t-induced (PDB:3ODU) | SDF-1α-induced (PDB: 8U4O) | None |
| 1 | IT1t-induced (PDB:3ODU) | SDV1a-induced (PDB: 9UPU) | None |
| 1 | AMD3100-induced (PDB:8ZPN) | AMD3100-induced (PDB: 8U4P) | None |
| 2 | IT1t-induced (PDB:3ODU) | SDF-1α-induced (PDB: 8U4O) | W94 or W94_E288 sidechain |
| 68 | IT1t-induced (PDB:3ODU) | SDF-1α-induced (PDB: 8U4O) | Alanine mutations |
| 1 | HL82624-induced (early stage) | SDF-1α-induced (PDB: 8U4O) | None |
| 1 | HL82624-induced (late stage) | SDF-1α-induced (PDB: 8U4O) | None |

**Table S2. 68 Residues selected for mutagenesis and their CG results**

| Mutation | Mutation Rationale | Motif | T1 | T3 | ΔT1 | ΔT3 |
|---|---|---|---|---|---|---|
| WT | | | 12.46 | 6.62 | | |
| N37A | [a]Structural variation | | 5.59 | 9.34 | -6.87 | 2.72 |
| L41A | Structural variation | | 6.26 | 7.50 | -6.20 | 0.88 |
| P42A | [b]Experimental mutation | | 4.86 | 6.37 | -7.60 | -0.25 |
| I44A | Experimental mutation | Pocket | 4.52 | 5.24 | -7.94 | -1.38 |
| Y45A | Experimental mutation | | 4.86 | 5.98 | -7.60 | -0.64 |
| G55A | Experimental mutation | | 4.29 | 7.54 | -8.17 | 0.92 |
| N56A | Structural variation | | 5.04 | 6.56 | -7.42 | -0.06 |
| T73A | Structural variation | | 5.07 | 4.67 | -7.39 | -1.95 |
| D74A | Structural variation | | 6.01 | 6.33 | -6.45 | -0.29 |
| K75A | Structural variation | [c]CCM | 4.13 | 5.81 | -8.33 | -0.81 |
| Y76A | Structural variation | | 7.63 | 5.77 | -4.83 | -0.85 |
| R77A | Structural variation | | 10.24 | 14.16 | -2.22 | 7.54 |
| H79A | Structural variation | | 7.82 | 9.66 | -4.64 | 3.04 |
| S81A | Structural variation | | 5.24 | 4.83 | -7.22 | -1.79 |
| D84A | Structural variation | | 9.07 | 7.82 | -3.39 | 1.20 |
| L86A | Experimental mutation | | 7.22 | 7.29 | -5.24 | 0.67 |
| F87A | Structural variation | | 4.86 | 8.93 | -7.60 | 2.31 |
| V88A | Experimental mutation | | 6.84 | 8.60 | -5.62 | 1.98 |
| D97A | Experimental mutation | Pocket | 5.77 | 10.75 | -6.69 | 4.13 |
| H113A | Structural variation | | 6.06 | 6.95 | -6.40 | 0.33 |
| Y116A | Experimental mutation | Pocket | 4.92 | 5.42 | -7.54 | -1.20 |
| N119A | Structural variation | | 10.63 | 5.90 | -1.83 | -0.72 |
| S122A | Experimental mutation | | 5.08 | 4.73 | -7.38 | -1.89 |
| I126A | Structural variation | CCM | 4.82 | 5.27 | -7.64 | -1.35 |
| F129A | Structural variation | | 6.26 | 12.11 | -6.20 | 5.49 |
| I130A | Structural variation | | 7.79 | 5.58 | -4.67 | -1.04 |
| S131A | Experimental mutation | | 5.95 | 6.11 | -6.51 | -0.51 |
| D133A | Structural variation | | 8.76 | 6.98 | -3.70 | 0.36 |
| R134A | Experimental mutation | DRY | 5.12 | 7.06 | -7.34 | 0.44 |
| N143A | Experimental mutation | | 5.51 | 5.95 | -6.95 | -0.67 |
| K149A | Structural variation | | 5.89 | 8.66 | -6.57 | 2.04 |
| L151A | Structural variation | | 5.70 | 9.55 | -6.76 | 2.93 |
| V156A | Structural variation | CCM | 4.63 | 12.40 | -7.83 | 5.78 |

| | | | | | | |
|---|---|---|---|---|---|---|
| W161A | Structural variation | | 4.36 | 7.26 | -8.10 | 0.64 |
| D171A | Structural variation | | 7.97 | 6.89 | -4.49 | 0.27 |
| C186A | Structural variation | | 5.38 | 6.59 | -7.08 | -0.03 |
| D187A | Experimental mutation | | 7.94 | 6.85 | -4.52 | 0.23 |
| F189A | Experimental mutation | | 5.13 | 5.34 | -7.33 | -1.28 |
| N192A | Experimental mutation | | 5.48 | 7.47 | -6.98 | 0.85 |
| W195A | Structural variation | | 5.13 | 6.42 | -7.33 | -0.20 |
| F201A | Structural variation | | 8.23 | 6.82 | -4.23 | 0.20 |
| H203A | Experimental mutation | | 5.10 | 5.16 | -7.36 | -1.46 |
| P211A | Experimental mutation | | 6.27 | 10.53 | -6.19 | 3.91 |
| Y219A | Experimental mutation | | 7.52 | 5.46 | -4.94 | -1.16 |
| L226A | Experimental mutation | | 5.04 | 4.27 | -7.42 | -2.35 |
| H228A | Structural variation | | 8.07 | 6.92 | -4.39 | 0.30 |
| R235A | Structural variation | | 7.66 | 4.07 | -4.80 | -2.55 |
| V242A | Experimental mutation | | 7.30 | 8.49 | -5.16 | 1.87 |
| I245A | Experimental mutation | | 7.38 | 6.65 | -5.08 | 0.03 |
| L246A | Experimental mutation | | 6.83 | 9.53 | -5.63 | 2.91 |
| F248A | Experimental mutation | | 9.47 | 6.82 | -2.99 | 0.20 |
| W252A | Experimental mutation | CWxP | 6.72 | 12.20 | -5.74 | 5.58 |
| Y255A | Structural variation | | 5.92 | 5.56 | -6.54 | -1.06 |
| Y256A | Structural variation | | 6.12 | 4.46 | -6.34 | -2.16 |
| S260A | Structural variation | | 5.70 | 6.51 | -6.76 | -0.11 |
| D262A | Experimental mutation | | 5.97 | 6.00 | -6.49 | -0.62 |
| F264A | Structural variation | | 7.93 | 8.09 | -4.53 | 1.47 |
| L267A | Experimental mutation | | 6.10 | 7.66 | -6.36 | 1.04 |
| H281A | Experimental mutation | | 6.81 | 4.41 | -5.65 | -2.21 |
| K282A | Experimental mutation | | 8.82 | 6.19 | -3.64 | -0.43 |
| W283A | Structural variation | | 6.29 | 11.96 | -6.17 | 5.34 |
| S285A | Experimental mutation | | 5.68 | 6.62 | -6.78 | 0.00 |
| I286A | Experimental mutation | | 5.58 | 5.39 | -6.88 | -1.23 |
| E288A | Experimental mutation | Pocket | 4.60 | 5.95 | -7.86 | -0.67 |
| F292A | Experimental mutation | | 9.27 | 5.43 | -3.19 | -1.19 |
| C295A | Structural variation | | 9.36 | 6.56 | -3.10 | -0.06 |
| Y302A | Experimental mutation | NPxxY | 7.68 | 10.47 | -4.78 | 3.85 |
| F304A | Structural variation | | 8.32 | 6.91 | -4.14 | 0.29 |

[a]Structural variation: Residues exhibiting significant conformational differences between the active and antagonist-bound states;

[b]Experimental mutation: Mutations reported in DOI: 10.1073/pnas.1601278113[5];

[c]CCM：Cholesterol consensus motif[6].

***Targeted Molecular Dynamics Simulations:*** Prior to generating the intermediate states for a given CXCR4 activation process, the corresponding refined endpoint structures were subjected to 5,000 energy minimization steps using the steepest-descent method to relieve close contacts. These minimizations were performed in the gas phase, with the temperature and step size set to 5 K and 0.1 fs, respectively, in preparation for subsequent calculations. Next, Targeted Molecular Dynamics (TMD) simulations[7] were employed to generate the intermediate states. In each case, the system was dragged from the initial (inactive) state toward the final (active) state through 151 successive mapping relaxation simulations, each comprising 10,000 steps, applying a harmonic dragging force of 100 $kcal \cdot mol^{-1} \cdot Å^{-2}$ to all heavy atoms. The step size was maintained as in the minimizations, while the temperature was raised to 300 K. From each TMD trajectory, 30 intermediate structures were extracted at evenly spaced intervals by selecting the last frame of every fifth mapping relaxation simulation, starting from the first relaxation, to provide representative conformations along the activation pathway.

***Relaxation and Equilibration Molecular Dynamics Simulations:*** All relaxation and equilibration MD simulations followed a common protocol. For each CXCR4 conformational state extracted from the TMD simulations, an implicit membrane was added to account for cellular membrane effects. The membrane consisted of a 50 × 60 × 44 Å particle grid along the Z-axis of the CXCR4 transmembrane bundle, with a spacing of 3 Å, and centered on the CXCR4 center of mass. Once the implicit membrane was added, the system was initially subjected to 2,000 energy minimization steps using the same parameters as described for previous minimizations. The system was then gradually heated from 5 to 300 K over 220 ps, while applying harmonic restraints that were progressively released. Specifically, during the first 60 ps, the temperature was increased to 100 K, and all protein atoms were constrained with a force constant of 10 $kcal \cdot mol^{-1} \cdot Å^{-2}$. In the next 60 ps, the temperature was raised to 250 K, and the restraints were reduced to 5 $kcal \cdot mol^{-1} \cdot Å^{-2}$. In the final heating step of 100 ps, the temperature reached 300 K, and restraints were applied only to protein backbone atoms using the same force constant. Finally, to obtain a representative ensemble of structures for each CXCR4 conformational state, a 100 ps equilibration MD simulation was performed, maintaining harmonic restraints on protein backbone atoms to preserve the conformation. From these trajectories, 10 evenly spaced structures were extracted for subsequent conformational free-energy calculations. The free energy of each intermediate we present in the main text is the average of the free energies of the 10

conformations.

***Coarse-Grained Calculations:*** To reliably estimate the conformational free energy ($\Delta G_{fold}$), the above extracted 10 geometries were then converted into the CG representation, in which the protein backbone remains in all-atom representation, while each side chain is represented as a simplified atom.[8] Prior to evaluating $\Delta G_{fold}$, the trimmed CG structures were submitted to a 20 ps equilibration under the same conditions as the previous MD simulations, with the exemption that the temperature was set to 150 K and no harmonic restraints were applied. The main feature of our Coarse-Grained (CG) model refined for membrane proteins, which has been successfully applied to GPCR[7, 9, 10], is its expression to evaluate $\Delta G_{fold}$:

$$\Delta G_{fold} = \Delta G_{side} + \Delta G_{main} + \Delta G_{sc.size} \tag{1}$$

$$= \Delta G_{side}^{elec} + \Delta G_{side}^{polar} + \Delta G_{side}^{hyd} + c_1 \Delta G_{side}^{VdW} + c_2 \Delta G_{solv}^{CG} + c_3 \Delta G_{HB}^{CG} + \Delta G_{sc.size}$$

Here, the total conformational free energy $\Delta G_{fold}$ consists of three terms: the sidechain term $\Delta G_{side}$, the main chain term $\Delta G_{main}$ and the so-called scaled size term $\Delta G_{sc.size}$, which can be broken down as specified in the second raw of Equation (1). $\Delta G_{side}$ accounts for side-chain contributions and comprises the first four breakdown terms: 1) $\Delta G_{side}^{elec}$, representing the free-energy change associated with solvation and charge–charge interactions of ionizable residues during folding; 2) $\Delta G_{side}^{polar}$, corresponding to the contribution of polar residues to the folding free energy; 3) $\Delta G_{side}^{hyd}$, describing the total hydrophobic contribution, including the implicit membrane effect; 4) $\Delta G_{side}^{VdW}$, representing the effective van der Waals interactions between simplified side chains. In contrast, $\Delta G_{main}$ includes contributions from backbone solvation ($\Delta G_{solv}^{CG}$) and hydrogen bonding ($\Delta G_{HB}^{CG}$). The last term, $\Delta G_{sc.size}$, accounts for the conformational entropy change arising from variations in the flexibility of the protein and its amino-acid side chains. The scaling coefficients $c_1$, $c_2$, $c_3$ were set to 0.10, 0.25 and 0.15, respectively. Finally, conformational free energy profiles were assembled by the CG conformational free energy of their constituent CXCR4 conformational states, whose energy corresponds to the $\Delta G_{fold}$ average of their 10 extracted geometries.

## 3. Binding free energy calculations of HF51116 and its fragments

***Methodology:*** The protein-dipole Langevin-dipole (PDLD) method[11] within the linear response approximation (LRA) framework (PDLD/S-LRA-2000) was employed to calculate the binding free energy ($\Delta G_{bind}$) of HF51116 and its fragments into CXCR4 with the aim of proving the hypothesis to rationally design small-molecule CXCR4 agonists. This approach evaluates the non-electrostatic term explicitly by a thermodynamic cycle, while the electrostatic term is evaluated by the LRA approach. Therefore, $\Delta G_{bind}$ is given the following expression:

$$\Delta G_{bind}=0.5\Delta G_{bind}^{elec}+C\Delta G_{bind}^{non\text{-}elec} \quad (2)$$

Where $\Delta G_{bind}$ refers to the total binding energy of ligand, $\Delta G_{bind}^{elec}$ and $\Delta G_{bind}^{non\text{-}elec}$ correspond to the electrostatic and non-electrostatic contributions of $\Delta G_{bind}$, respectively, and C is the scaling factor of this last contribution. The scaling factor C is not a universal constant, but is specific to the studied system, since it is influenced by the interplay between different thermodynamic contributions such as van der Waals interactions, hydrophobic effects, water penetration and configurational entropy changes[12]. Therefore, this factor is cavity-dependent. According to the following benchmark, an optimal C value of 0.2 has been determined for the CXCR4 orthosteric site, which allows reproducing the experimental value of the HF51116 binding free energy in this cavity.

***Benchmark:*** A benchmark procedure was carried out to determine the number of protein and water configurations required to achieve convergence of the binding free energy ($\Delta G_{bind}$). In addition, taking advantage of the experimental binding free energy of HF51116 ($\Delta G_{bind}^{HF51116}$), the optimal scaling factor C for the non-electrostatic contribution ($\Delta G_{bind}^{non\text{-}elec}$) in the CXCR4 orthosteric site was determined. Initially, the standard scaling factor (C = 0.25) for the non-electrostatic contribution was used for benchmarking purposes. The generated $\Delta G_{bind}^{HF51116}$ values correspond to averages over four independent HF51116–CXCR4 complexes. A fixed ratio of 10:1 between the number of protein and water configurations was tested, as convergence in water is significantly faster than in protein. **Table S3** summarizes $\Delta G_{bind}^{HF51116}$ and its individual contributions ($\Delta G_{bind}^{elec}$ and $\Delta G_{bind}^{non\text{-}elec}$) as a function of the number of sampled configurations, while **Figure S3** shows the convergence behavior of $\Delta G_{bind}^{HF51116}$. Based on the benchmark results, 50,000 and 5,000 configurations for protein and water,

respectively, were selected as optimal, providing a reliable balance between convergence and computational cost. Under these conditions, $\Delta G_{bind}$ values exhibited a statistical uncertainty of approximately 1 kcal/mol, supporting the robustness of the chosen sampling protocol. This provides a solid justification for the use of these parameters in all subsequent $\Delta G_{bind}$ calculations presented in this work.

**Table S3. Benchmark for different numbers of configurations in protein and water**

| Number of configurations in protein | Number of configurations in water | $\Delta G_{bind}^{elec}$ (kcal/mol) | [a]$\Delta G_{bind}^{non-elec}$ (kcal/mol) | $\Delta G_{bind}^{HF51116}$ (kcal/mol) |
|---|---|---|---|---|
| 100 | 10 | -8.25 ± 0.91 | 2.78 ± 1.09 | -5.47 ± 1.52 |
| 250 | 25 | -8.23 ± 0.82 | 0.42 ± 0.73 | -7.81 ± 0.37 |
| 500 | 50 | -8.59 ± 1.13 | 0.19 ± 0.97 | -8.40 ± 1.65 |
| 750 | 75 | -8.50 ± 0.83 | -0.29 ± 1.14 | -8.79 ± 1.29 |
| 1000 | 100 | -7.96 ± 0.96 | 0.83 ± 0.71 | -7.13 ± 1.41 |
| 2500 | 250 | -7.61 ± 0.84 | -0.31 ± 0.59 | -7.92 ± 1.08 |
| 5000 | 500 | -8.20 ± 0.27 | -0.71 ± 0.20 | -8.91 ± 0.24 |
| 7500 | 750 | -9.18 ± 0.78 | -1.34 ± 0.47 | -10.51 ± 0.80 |
| 10000 | 1000 | -8.94 ± 0.86 | -1.20 ± 0.72 | -10.14 ± 0.83 |
| 25000 | 2500 | -9.51 ± 0.65 | -2.14 ± 0.34 | -11.64 ± 0.85 |
| 50000 | 5000 | -9.40 ± 0.72 | -1.88 ± 0.51 | -11.28 ± 0.41 |
| 75000 | 7500 | -9.27 ± 1.03 | -3.12 ± 0.17 | -12.39 ± 1.07 |
| 100000 | 10000 | -9.63 ± 0.52 | -2.32 ± 0.48 | -11.95 ± 0.75 |

[a]The standard scaling factor (C=0.25) of $\Delta G_{bind}^{non-elec}$ was employed.

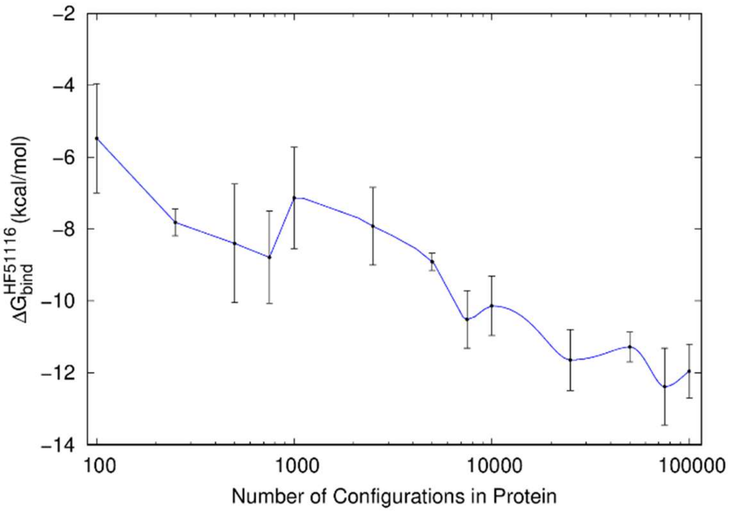


**Figure S3.** $\Delta G_{bind}^{HF51116}$ as a function of number of configurations in protein. Errors bars indicate the $\Delta G_{bind}^{HF51116}$ uncertainty, which was estimated by the standard error of the mean (SEM) of four calculations.

$\Delta G_{bind}$ ***calculations:*** The simulation setup for $\Delta G_{bind}$ calculations was similar to that used to generate the relaxed complexes described above, with several modifications: The cryo-EM structure of CXCR4 bound to HF51116 in the antagonist state (PDB ID: 8ZPL) was used as the starting system. In the PDLD/S-LRA calculations, the system was divided into two regions: Region 1, corresponding to the ligand whose binding free energy was evaluated, and Region 2, consisting of the surrounding environment contributing to ligand binding. For each system, the complex was solvated using a 20 Å water sphere centered on the ligand and described by the surface-constrained all-atom solvent (SCAAS) model[13] implemented in MOLARIS-XG, with long-range electrostatic interactions treated using the local reaction field (LRF) method. Four geometries were extracted from the equilibration MD simulation for subsequent $\Delta G_{bind}$ calculations. A total of 50,000 configurations for the protein environment and 5,000 for the aqueous reference state were sampled to ensure convergence of $\Delta G_{bind}$, consistent with previous and current benchmarks.[10] Finally, $\Delta G_{bind}$ of the ligand corresponds to the average obtained from the four extracted complexes (**Table S4**). When HF51116 fragments were considered, harmonic restraints (5 $kcal \cdot mol^{-1} \cdot Å^{-2}$) were applied to the ligand to prevent their displacement within the CXCR4 orthosteric binding site. The partial charges of HF51116 and its fragments were derived from DFT calculations at the B3LYP/def2-TZVP level, followed by RESP fitting[14] based on the electrostatic potential calculated using Gaussian.

**Table S4. $\Delta G_{bind}$ for HF51116 and its fragments**

| **Compound** | **$^{a}\Delta G_{bind}$ (kcal/mol)** |
|---|---|
| HF51116 | -10.90 ± 0.44 |
| F01 | -13.55 ± 1.52 |
| F02 | -3.83 ± 0.61 |

[a]The results employed the optimal setting for the scaling factor C (C=0.20) and the number of configurations in protein and water (50,000 and 5,000, respectively).

## 4. Molecular docking and molecular dynamics of HL82624-CXCR4 system

***Induced Fit Docking:*** The initial HL82624–CXCR4 complex used for Molecular dynamics (MD) simulations was generated using the Induced Fit Docking (IFD) module implemented in the Maestro package (Schrödinger Inc.). The inactive CXCR4 structure (PDB ID: 3ODU) was used as the receptor. To define the binding site, 3ODU was structurally aligned with the HF51116-bound inactive CXCR4 structure (PDB ID: 8ZPL), and a docking grid was centered on the HF51116 binding region with a box size of 20 Å. A core-constrained docking protocol was applied, using the central phenyl ring

of HF51116 as the reference position. The "trim side chains" option was enabled, and residue W94 was selected as the induced-fit residue to allow conformational adaptation during docking. Among the generated poses, the one that successfully induced W94 into its active-like conformation and exhibited a key pharmacophore (Lys-Pro) binding mode similar to that of SDF-1α was selected as the starting structure for subsequent MD simulations. The same induced fit docking protocol was applied to HL73024. The predicted binding modes of HL82624 and HL73024 are shown in **Figure S4**.

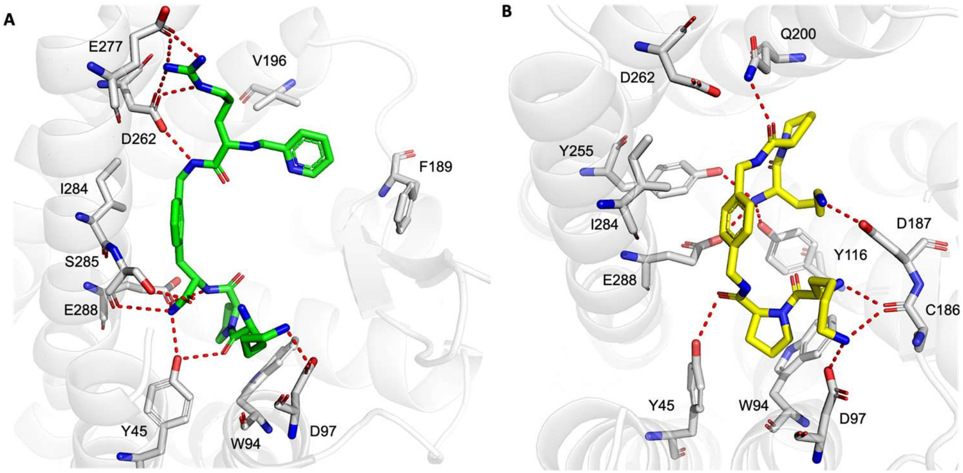


**Figure S4.** Predicted binding modes of HL82624 (A) and HL73024 (B) obtained from induced-fit docking simulations.

***Molecular dynamics simulations:*** The generated HL82624-CXCR4 complex was embedded into a lipid bilayer using the membed method,[15] with the membrane orientation determined by the PPM web server.[16] The membrane consisted of 118 1-Palmitoyl-2-oleoyl-sn-glycero-3-phosphocholine (POPC) molecules forming a symmetric bilayer. The system was solvated with approximately 5000 water molecules and neutralized by the addition of 9 chloride ions. The Amber force field ff14SB was employed to describe protein atoms, while CHARMM36 force field and TIP3P model were used for POPC and water molecules, respectively. Specific parameters of HF82624 were developed here. The B3LYP/def2-TZVP level of theory was employed to compute the Merz–Kollman atomic charges of HF82624, and were then fitted using the RESP procedure.[14] According to our chemist experience, HL82624 was modeled in its +4 charge state.

Regarding the MD simulation protocol, the system was first subjected to 10,000 steps of energy minimization using the conjugate gradient algorithm to remove unfavorable contacts. During minimization, the maximum step size and force tolerance were set to 0.1 Å and 10 kcal $mol^{-1}$ $Å^{-1}$, respectively. Subsequently, all-atom MD simulations were performed under periodic boundary conditions. Position restraints were applied to the

heavy atoms of protein and water with a force constant of 10 kJ mol$^{-1}$ Å $^{-2}$ during the heating and pressurization of the system, whose length was 200 ps. Temperature was controlled to 303 K using the V-rescale thermostat, with separate coupling groups defined for the protein–ligand complex, POPC membrane, and solvent/ions. Pressure was maintained semi-isotropically at 1 bar using the Parrinello–Rahman barostat. This was followed by a 200 ns equilibration MD simulation in the NPT ensemble and a subsequent 300 ns production MD simulation. To preserve ligand's binding mode within the orthosteric site, position restraints were applied to the heavy atoms of the ligand at those stages with a force constant of 10 kJ mol$^{-1}$ Å $^{-2}$. From this trajectory, representative structures corresponding to the early and late stages of the production run were extracted to generate structural models of the HF82624-induced CXCR4 inactive state used in the conformational free energy calculations. These temporally distant conformations were selected to enhance conformational diversity and to improve the exploration of activation pathways. Long-range electrostatic interactions were treated using the particle mesh Ewald (PME) method with a cutoff of 10 Å, while van der Waals interactions were described using a Lennard–Jones potential with the same cutoff. All bonds involving hydrogen atoms were constrained using LINCS algorithms, allowing a time step of 2 fs throughout the simulations.

## Biological assay details

### 1. 12G5 Competitive Binding Assay

For the 12G5 competitive binding method, Sup-T1 cells were harvested from the culture of RPMI 1640 medium (containing 10% FBS, 100 IU penicillin, and 0.1 mg/mL streptomycin) and washed with FACS buffer (0.5% BSA and 0.05% $NaN_3$ in PBS), and then seeded into V-bottom 96-well plate at a density of 1 × $10^6$ cells per well. Various concentrations of HL82624 were added to the wells along with the anti-CXCR4 antibody 12G5 (250 ng/mL, BD Biosciences, USA). Following incubation on ice for 40 min, cells were washed twice by centrifugation with FACS buffer and subsequently incubated with a secondary IgG-FITC antibody (Sigma-Aldrich, USA) for 30 min on ice. After incubation, cells were washed twice with FACS buffer by centrifugation. The fluorescence intensity ($485_{EX}$ / $535_{EM}$) was measured using a Synergy™ 2 microplate reader (BioTek, USA).

## 2. Cell Migration Assay

A high-throughput (HTS) Transwell 96-well plate with 5 μm pore size (Coring, USA) was used for the cell migration assay. Sup-T1 cells (ATCC, USA) were cultured in RPMI 1640 medium containing 10% FBS, 100 IU penicillin, and 0.1 mg/mL streptomycin. Cells were harvested and washed twice by centrifugation with assay buffer of RPMI1640 medium containing 0.5% BSA, and then resuspended in assay buffer at a concentration of $2.67 \times 10^7$ cells/mL. The cell suspension was added to the upper chambers of Transwell plate. Various concentrations of HL82624, HL73024, and the positive control SDF-1α were prepared in 180 μL of assay buffer and added to the lower chambers. Lower chambers containing assay buffer alone (without SDF-1α or compounds) served as background controls. The plate was placed in the cell incubator for 3 hr at 37°C. After incubation, the upper chambers were carefully removed. Then, 36 μL of CellTiter 96 reagent (Promega, USA) was added to each well of the lower chambers. Following a 4 hrs incubation at 37°C, the number of migrated cells was quantified by measuring absorbance at 490 nM using a Synergy™ 2 microplate reader (BioTek, USA).

# Synthetic details

## 1. Synthetic route

Target compounds **HL82624** and **HL73024** were synthesized as described in Scheme 1. Compound **1** undergoes an amide formation reaction with DMF to generate intermediate **2**. Subsequently, the BOC protecting group of **2** is removed under acidic conditions, followed by a coupling reaction with **A1** to yield intermediate **4**. Intermediate **4** is then transformed into intermediate **7** through reduction, a second round of amide formation, and removal of the Fmoc protecting group. Finally, target product **HL82624** is obtained through reductive amination and removal of any remaining protecting groups. Concurrently, an excess of compound **9** undergoes two coupling reactions with a diamine, followed by the removal of all protecting groups to afford compound **HL73024**.

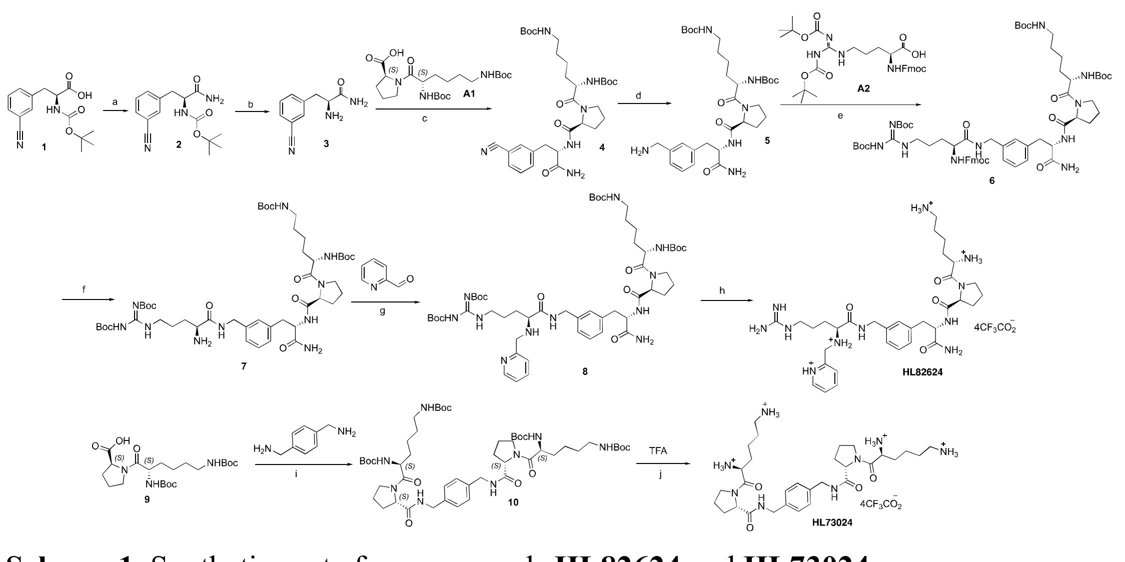

**Scheme 1**. Synthetic route for compounds **HL82624** and **HL73024**

[a]Reagents and conditions: (a) HATU, DIPEA, DMF, rt, 2 h; (b) HCl in 1,4-dioxane, 0 °C→20 °C, 3 h; (c) HATU, DIPEA, DMF, rt, 2 h; (d) Pd/C, $H_2$, $NH_3$ $H_2O$, $CH_3OH$, rt, 16 h; (e) EDCI, HOBT, DCM, 0 °C, 1 h; (f) Piperidine, DCM, rt, 1 h; (g) $NaBH_3CN$, HAC, MeOH, rt, 16 h; (h) TFA, rt, 16 h; (i) 1,4-phenylenedimethanamine, HATU, DIPEA, DCM, 0 °C→rt, 3 h; (j) TFA/DCM, rt, 1 h.

## 2. Synthetic details

Commercially available reagents and solvents were used directly without further purification. The high resolution MS (HRMS) of target compounds were analyzed by using Waters Xevo G2 QTof. $^{1}$H NMR and $^{13}$C NMR spectra were obtained on the Bruker Ascend™ 500. The chemical shifts (δ) are reported in parts per million (ppm) using suitable deuterated NMR solvents in reference to tetramethyl silane (TMS) at 0 ppm. Multiplicities are defined as follows: s (singlet), d (doublet), t (triplet), q (quartet), dd (doublet of doublets), and m (multiplets). The reaction was monitored by TLC (silica gel GF254) or HPLC. The compounds were purified by Flash chromatography packed with silica gel (silica gel 100-200 mesh or 200-300 mesh).

**Tert-butyl (S)-(1-amino-3-(3-cyanophenyl)-1-oxopropan-2-yl)carbamate (2)**

To a mixture of (S)-2-((tert-butoxycarbonyl)amino)-3-(3-cyanophenyl)propanoic acid (500 mg, 1.72 mmol), DIPEA (891 mg, 6.9 mmol), HATU (982 mg, 2.59 mmol) in DMF (5 mL) was added $NH_4Cl$ (922 mg, 17.24 mmol) at 0 °C. The resulting mixture was stirred at 20 °C for 2 h. The reaction mixture was poured to saturated $NaHCO_3$ (10 mL) and extracted with DCM (20 mL x 4). The combined organic layers were washed with brine (10 mL x 1), dried over anhydrous $Na_2SO_4$, concentrated. The crude product was purified by column chromatography on silica gel (PE:EA = 40:60) to give compound **2** (500 mg, 72.7 % Purity , yield = 73% ) as yellow solid. MS (ES): *m/z* = 234 $[M+H-56]^+$.

**(S)-2-Amino-3-(3-cyanophenyl)propanamide (3)**

To a mixture of compound **2** (500 mg,1.73 mmol) in dioxane (10 mL) was added 4M HCl/Dioxane (4mL) at 0°C. The solution was stirred at 20 °C for 3 hours. The organic was concentrated in vacuo to give compound **3** (370 mg, 64% Purity, yield = 100%) as yellow oil. MS (ES): *m/z* = 190 $[M+H]^+$.

**Di-tert-butyl ((S)-6-(((S)-1-(((S)-1-amino-3-(3-cyanophenyl)-1-oxopropan-2-yl)amino)-1-oxopropan-2-yl)(methyl)amino)-6-oxohexane-1,5-diyl)dicarbamate (4)**

To a mixture of compound **3** (270 mg, 1.43 mmol), DIPEA (739 mg, 5.71 mmol), HATU (814 mg, 2.14 mmol) in DMF (2 mL) was added BOC-LYS(BOC)-PRO-OH (633 mg, 1.43 mmol) at 0 °C and the mixture was stirred at 20 °C for 2 h. The reaction mixture was poured to saturated $NaHCO_3$ (10 mL) and extracted with DCM (50 mL x 4). The combined organic layers were washed with brine (20 mL x 1), dried over anhydrous $Na_2SO_4$, concentrated. The crude product was purified by column chromatography on silica gel (25 g, PE:EA = 40:60) to give compound **4** (423 mg, 96% Purity, yield=75%) as yellow solid. MS (ES): *m/z* = 615.5 $[M+H]^+$. $^1$H NMR (500 MHz, DMSO-$d_6$) δ 7.83 (d, $J$ = 8.2 Hz, 1H), 7.72 – 7.61 (m, 2H), 7.57 (d, $J$ = 8.0 Hz, 1H), 7.47 (t, $J$ = 7.7 Hz, 1H), 7.20 (d, $J$ = 53.9 Hz, 2H), 7.01 – 6.62 (m, 2H), 4.42 (d, $J$ = 5.0 Hz, 1H), 4.26 (d, $J$ = 4.1 Hz, 1H), 4.13 (s, 1H), 3.53 (s, 2H), 3.08 (d, $J$ = 4.8 Hz, 1H), 2.89 (dd, $J$ = 13.7, 8.5 Hz, 3H), 1.97 – 1.89 (m, 1H), 1.88 – 1.78 (m, 2H), 1.65 (dd, $J$ = 11.6, 5.2 Hz, 1H), 1.34 (dd, $J$ = 24.2, 9.8 Hz, 24H). $^{13}$C NMR (126 MHz, DMSO-$d_6$) δ 172.20, 155.49, 134.32, 132.75, 130.05, 129.20, 77.91, 77.30, 59.68, 53.07, 39.15, 36.58, 30.37, 29.24, 28.72, 28.21, 24.44, 22.55.

**Di-tert-butyl ((S)-6-((S)-2-(((S)-1-amino-3-(3-(aminomethyl)phenyl)-1-oxopropan-2-yl)carbamoyl)pyrrolidin-1-yl)-6-oxohexane-1,5-diyl)dicarbamate (5)**

A mixture of compound **4** (410 mg, 0.67 mmol) and Pd/C (200 mg) in MeOH (10 mL) was stirred for 16 h under hydrogen atmosphere. The mixture was filtered through Celite. The filtrate was concentrated and the crude product was purified by column chromatography on silica gel (25 g, PE:EA = 60:40) to give compound **5** (144 mg, 85% Purity, yield=30%) as white solid. MS (ES): *m/z* = 619.5 $[M+H]^+$.

**To synthesize the compound 6**

A mixture of compound **5** (100 mg, 0.16 mmol) in DCM (5 mL) was added EDCI (96 mg, 0.16 mmol,1.2 equiv), HOBt (26 mg, 0.19 mmol, 1.2 equiv) at 0 °C and stirred at 20 °C for 1 h. To above solution was added FMOC-ARG(BOC)$_2$-OH (96 mg, 0.16 mmol) at 0 °C and the mixture was stirred at 20 °C for 2 h. The reaction mixture was poured to saturated $NaHCO_3$ (10 mL) and extracted with DCM (20 mL x 4). The combined organic layers were washed with brine (10 mL x 1), dried over anhydrous

$Na_2SO_4$, concentrated. The crude product was purified by column chromatography on silica gel (25 g, PE:EA = 40:60) to give the product (65 mg, 100% Purity ,yield=40%) as yellow solid. MS (ES): *m/z* = 1198 $[M+H]^+$. $^1H$ NMR (400 MHz, Methanol-$d_4$) δ 7.78 (d, $J$ = 7.5 Hz, 2H), 7.71 – 7.61 (m, 2H), 7.37 (t, $J$ = 7.4 Hz, 2H), 7.28 (t, $J$ = 7.4 Hz, 2H), 7.24 – 7.04 (m, 4H), 4.66 – 4.17 (m, 8H), 3.62 (s, 2H), 3.35 (s, 2H), 3.01 (d, $J$ = 6.7 Hz, 4H), 2.07 (dt, $J$ = 13.2, 6.2 Hz, 1H), 1.97 – 1.74 (m, 4H), 1.73 – 1.36 (m, 45H). $^{13}C$ NMR (101 MHz, Methanol-$d_4$) δ 174.39, 173.24, 172.58, 171.58, 163.18, 157.10, 156.60, 156.22, 152.78, 144.01, 143.74, 141.24, 138.49, 137.44, 128.80, 127.61, 127.40, 126.80, 125.38, 124.89, 119.53, 83.08 , 79.08, 78.42, 66.46, 60.14, 55.06, 54.23, 52.36, 48.26, 46.98, 42.50, 39.90, 39.61, 36.94, 30.71 , 29.67, 28.48, 27.41, 26.89, 25.51, 24.56, 22.57, 19.49.

**To synthesize the compound 7**

To a solution of compound **6** (65 mg, 0.054 mmol) in DCM (10 mL) was added piperidine (1 mL). The mixture was stirred at 20 °C for 1 h. The reaction mixture was concentrated. The crude product (150 mg) as yellow solid which was used in next step without purified. MS (ES): *m/z* = 977 $[M+H]^+$.

**To synthesize the compound 8**

To a solution of picolinaldehyde (10 mg, 0.09 mmol) in MeOH (5 mL) was added compound **7** (110 mg, 0.11 mmol) ), AcOH (13.5 mg, 0.23 mmol) and $NaBH_3CN$ (14 mg, 0.23 mmol) at 0 °C. The mixture was stirred at 20 °C for 16 h. The reaction mixture was added water (10 mL) and extracted with EtOAc (10 mL x 3). The organic layers were combined, washed with brine, dried over $Na_2SO_4$, concentrated. The crude product was purified by Pre-HPLC (water (0.05%TFA)/MeCN) to give the product as solution without freeze-drying. MS (ES): *m/z* = 533.8 $[M/2+H]^+$.

**To synthesize the compound HL82624**

To the above solution was added TFA (10 mL) at 0 °C. The mixture was stirred at 40 °C for 16 h. The reaction mixture was concentrated under reduced pressure. The residue was transferred to vial with (water/MeCN) and lyophilized to give the expected product (19 mg, 100% Purity). MS (ES): *m/z* = 667.5 $[M+H]^+$. $^1H$ NMR (500 MHz, Methanol-$d_4$) δ 8.62 (d, $J$ = 4.3 Hz, 1H), 7.86 (t, $J$ = 7.6 Hz, 1H), 7.42 (t, $J$ = 9.1 Hz, 2H), 7.32 – 7.07 (m, 4H), 4.52 – 4.19 (m, 6H), 4.01 (d, $J$ = 6.2 Hz, 1H), 3.78 – 3.55 (m, 2H), 3.22 (t, $J$ = 6.7 Hz, 2H), 3.09 (ddd, $J$ = 21.6, 13.8, 7.1 Hz, 2H), 2.96 (d, $J$ = 6.9 Hz, 2H), 2.21 (d, $J$ = 5.7 Hz, 1H), 2.03 (s, 3H), 2.00 – 1.84 (m, 4H), 1.80 – 1.43 (m, 7H), 0.90 (t, $J$ = 6.8 Hz, 1H). $^{19}F$ NMR (376 MHz, Methanol-$d_4$) δ -77.04 (s).

**To synthesize the compound 10**

To a solution of compound **9** (500 mg, 1.12 mol) in DCM (15 mL) was added 1,4-phenylenedimethanamine (61 mg, 0.448 mmol), HATU (640 mg, 1.69 mmol) and DIPEA (440 mg, 3.38 mmol) at 0 ºC. The mixture stirred at room temperature for 3 h.

The mixture was quenched with $H_2O$ (20 mL) and extracted with DCM (30 mL x 2). The combined organic phase was washed with aq. HCl (1N) and brine, dried over $Na_2SO_4$, filtered and concentrated. The residue was purified by prep-HPLC (water (10 mM $NH_4HCO_3$)/ACN) to give compound **10** (150 mg, yield 13.5%) as white solid. MS (ESI): *m/z*= 987.6 $[M+H]^+$. $^1H$ NMR (400 MHz, $CDCl_3$) δ 7.34-7.18 (m, 4H), 5.44-5.31 (m, 2H), 5.08 (s, 2H), 4.61-4.59 (m, 3H), 4.48-4.20 (m, 5H), 3.78-3.51 (m, 4H), 3.02-2.94 (m, 4H), 2.39-2.27 (m, 2H), 2.21-2.08 (m, 2H), 2.04-1.81(m, 12H), 1.52-1.36 (m, 40H). $^{13}C$ NMR (101 MHz, $CDCl_3$) δ 172.58, 171.19, 156.26, 155.66, 137.35, 127.69, 79.80, 79.00, 77.37, 77.06, 76.74, 59.98, 51.81, 47.41, 43.04, 40.00, 32.28, 29.45, 28.42, 27.54, 25.18, 22.28.

**To synthesize the compound HL73024**

A mixture of compound **10** (70 mg, 0.05 mmol) in TFA/DCM(1/2) (10 mL) was stirred at room temperature for 1 h. The mixture was concentrated and the residue was purified by prep-HPLC (water (0.05%TFA)/MeCN) to give (2S,2'S)-N,N'-(1,4-phenylenebis(methylene))bis(1-(L-lysyl)pyrrolidine-2-carboxamide) TFA salt (30 mg, yield 40.5%) as colorless semisolid. MS (ESI): *m/z* = 587 $[M+H]^+$.$^1H$ NMR (400 MHz, $D_2O$) δ 7.15 (s, 4H), 4.32 (dd, *J* = 16.8, 8.9 Hz, 4H), 4.27-4.09 (m, 4H), 3.72-3.55 (m, 2H), 3.53-3.39 (m, 2H), 2.91-2.73 (m, 4H), 2.30-2.09 (m, 2H), 1.99-1.69 (m, 10H), 1.64-1.46 (m, 4H), 1.42-1.19 (m, 4H).$^{13}C$ NMR (126 MHz, $D_2O$) δ 173.61, 168.17, 163.05, 162.77, 136.88, 127.43, 117.50, 115.18, 60.90, 51.61, 48.03, 42.64, 38.95, 29.36, 26.40, 24.72, 20.75.$^{19}F$ NMR (376 MHz, $D_2O$) δ -75.59.

# III. $^1$H NMR AND $^{13}$C NMR SPECTRA OF TARGET COMPOUND

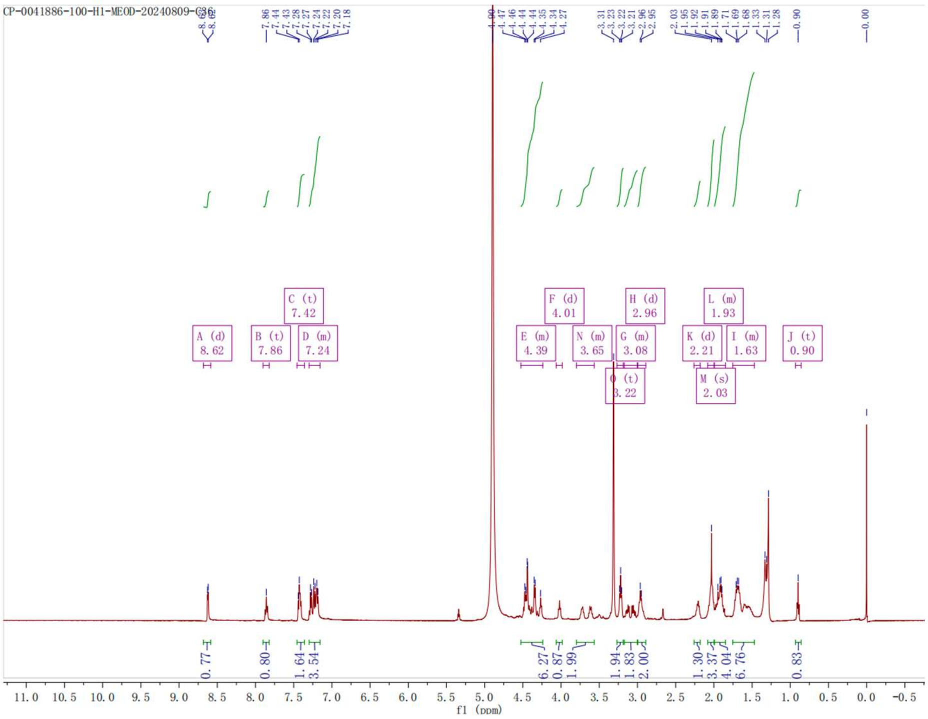


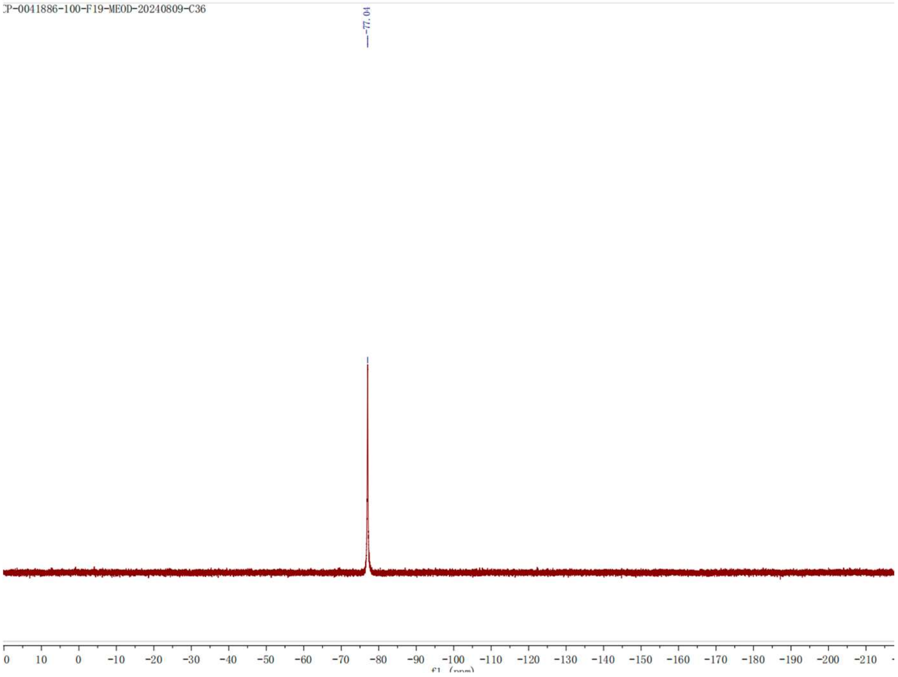


**Figure S5.** $^1$H NMR and $^{19}$F NMR of **HL82624**

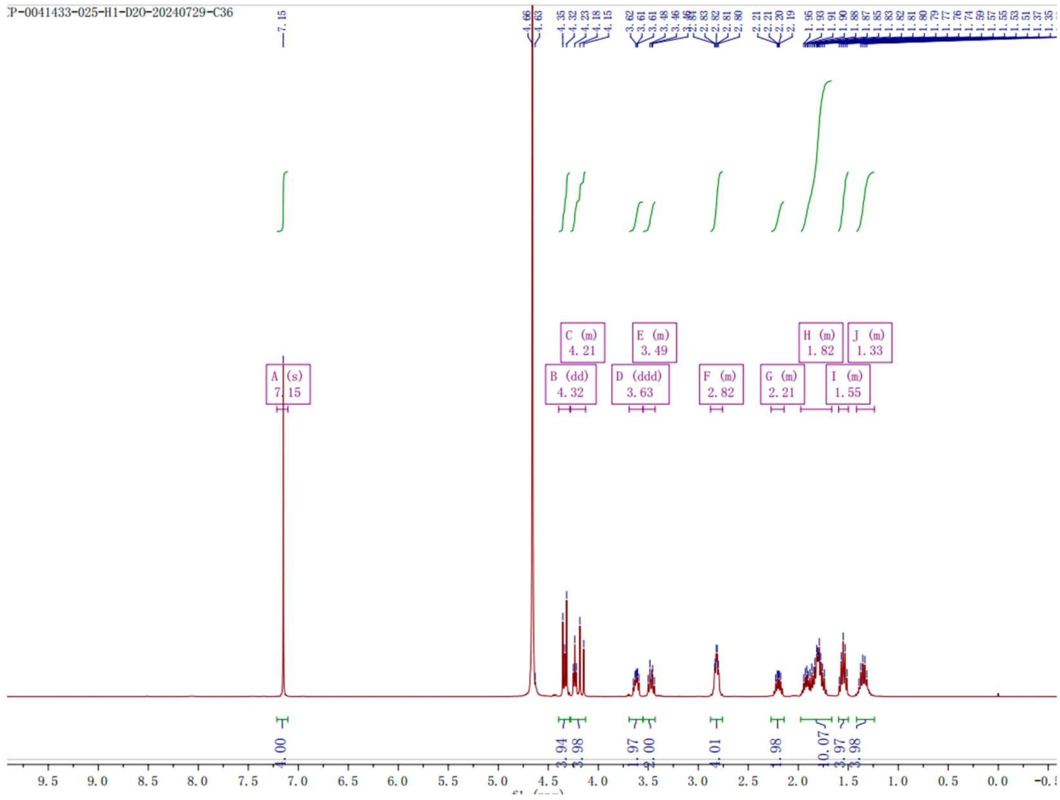
CP-0041433-025-H1-D20-20240729-C36
A (s) 7.15
B (dd) 4.32
C (m) 4.21
D (ddd) 3.63
E (m) 3.49
F (m) 2.82
G (m) 2.21
H (m) 1.82
I (m) 1.55
J (m) 1.33
f1 (ppm)

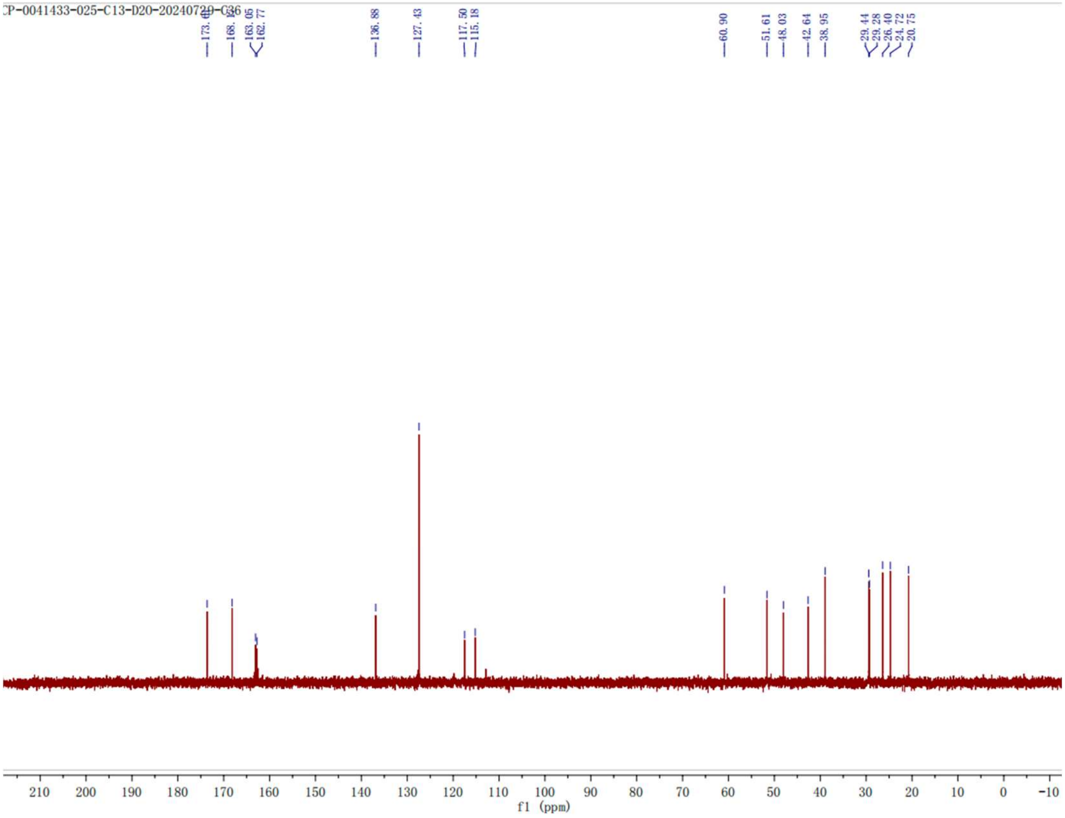
CP-0041433-025-C13-D20-20240729-C36
136.88
127.43
117.50
115.18
60.90
51.61
48.03
42.64
38.95
29.44
29.28
26.40
24.72
20.75
f1 (ppm)

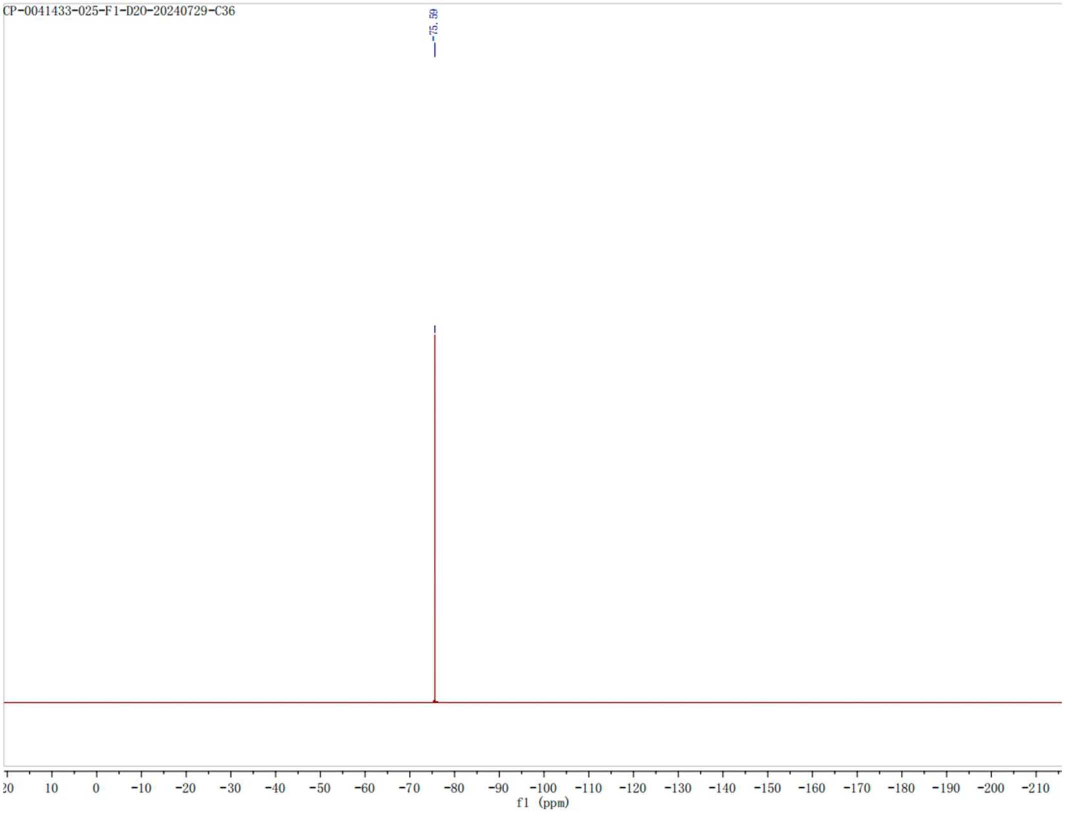


**Figure S6.** $^{1}$H NMR, $^{13}$C NMR, and $^{19}$F NMR of **HL73024**

# IV. SUPPLEMENTARY REFERENCES


(1) Warshel, A.; Chu, Z.; Villa, J.; Strajbl, M.; Schutz, C.; Shurki, A.; Vicatos, S.; Plotnikov, N.; Schopf, P. Molaris-XG, v 9.15. *University of Southern California: Los Angeles* **2012**.

(2) Abraham, M. J.; Murtola, T.; Schulz, R.; Páll, S.; Smith, J. C.; Hess, B.; Lindahl, E. GROMACS: High performance molecular simulations through multi-level parallelism from laptops to supercomputers. *SoftwareX* **2015**, *1*, 19-25.

(3) Pettersen, E. F.; Goddard, T. D.; Huang, C. C.; Couch, G. S.; Greenblatt, D. M.; Meng, E. C.; Ferrin, T. E. UCSF Chimera—a visualization system for exploratory research and analysis. *J. Comput. Chem.* **2004**, *25* (13), 1605-1612.

(4) Sham, Y. Y.; Chu, Z. T.; Warshel, A. Consistent calculations of p K a's of ionizable residues in proteins: semi-microscopic and microscopic approaches. *J. Phys. Chem. B* **1997**, *101* (22), 4458-4472.

(5) Stephens, B. S.; Ngo, T.; Kufareva, I.; Handel, T. M. Functional anatomy of the full-length CXCR4-CXCL12 complex systematically dissected by quantitative model-guided mutagenesis. *Sci. Signal.* **2020**, *13* (640), eaay5024.

(6) Trzaskowski, B.; Latek, D.; Yuan, S.; Ghoshdastider, U.; Debinski, A.; Filipek, S. Action of molecular switches in GPCRs-theoretical and experimental studies. *Curr. Med. Chem.* **2012**, *19* (8), 1090-1109.

(7) Bai, C.; Wang, J.; Mondal, D.; Du, Y.; Ye, R. D.; Warshel, A. Exploring the activation process of the β2AR-Gs complex. *J. Am. Chem. Soc.* **2021**, *143* (29), 11044-11051.
(8) Kamerlin, S. C.; Vicatos, S.; Dryga, A.; Warshel, A. Coarse-grained (multiscale) simulations in studies of biophysical and chemical systems. *Annu. Rev. Phys. Chem.* **2011**, *62* (1), 41-64.
(9) Zhu, X.; Luo, M.; An, K.; Shi, D.; Hou, T.; Warshel, A.; Bai, C. Exploring the activation mechanism of metabotropic glutamate receptor 2. *Proc. Natl. Acad. Sci. USA* **2024**, *121* (21), e2401079121.
(10) Cruz, A.; Warshel, A. Efficient Characterization of GPCRs Allosteric Modulation: Application to the Rational Design of De Novo S1PR1 Allosteric Modulators. *J. Chem. Inf. Model.* **2025**, *65* (16), 8637-8652.
(11) Singh, N.; Warshel, A. Absolute binding free energy calculations: On the accuracy of computational scoring of protein–ligand interactions. *Proteins: Struct. Funct. Bioinform.* **2010**, *78* (7), 1705-1723.
(12) Sham, Y. Y.; Chu, Z. T.; Tao, H.; Warshel, A. Examining methods for calculations of binding free energies: LRA, LIE, PDLD-LRA, and PDLD/S-LRA calculations of ligands binding to an HIV protease. *Proteins* **2000**, *39*, 393-407.
(13) King, G.; Warshel, A. A surface constrained all-atom solvent model for effective simulations of polar solutions. *J. Chem. Phys.* **1989**, *91* (6), 3647-3661.
(14) Bayly, C. I.; Cieplak, P.; Cornell, W.; Kollman, P. A. A well-behaved electrostatic potential based method using charge restraints for deriving atomic charges: the RESP model. *J. Phys. Chem* **1993**, *97* (40), 10269-10280.
(15) Wolf, M. G.; Hoefling, M.; Aponte-Santamaría, C.; Grubmüller, H.; Groenhof, G. g_membed: Efficient insertion of a membrane protein into an equilibrated lipid bilayer with minimal perturbation. *J. Comput. Chem.* **2010**, *31* (11), 2169-2174.
(16) Lomize, M. A.; Pogozheva, I. D.; Joo, H.; Mosberg, H. I.; Lomize, A. L. OPM database and PPM web server: resources for positioning of proteins in membranes. *Nucleic Acids Res.* **2012**, *40* (D1), D370-D376.
